# Neutral and adaptive loci reveal fine‐scale population structure in *Eleginops maclovinus* from north Patagonia

**DOI:** 10.1002/ece3.9343

**Published:** 2022-10-03

**Authors:** Cristian B. Canales‐Aguirre, Wesley A. Larson, Garrett J. McKinney, C. Eliza Claure, J. Dellis Rocha, Santiago G. Ceballos, María I. Cádiz, José M. Yáñez, Daniel Gomez‐Uchida

**Affiliations:** ^1^ Centro i~mar, Universidad de Los Lagos Puerto Montt Chile; ^2^ Núcleo Milenio INVASAL Concepción Chile; ^3^ National Oceanographic and Atmospheric Administration, National Marine Fisheries Service, Alaska Fisheries Science Center Auke Bay Laboratories Juneau Alaska USA; ^4^ Washington Department of Fish and Wildlife Seattle Washington USA; ^5^ Centro Austral de Investigaciones Científicas (CADIC‐CONICET) Ushuaia Tierra del Fuego Argentina; ^6^ Universidad Nacional de Tierra del Fuego (ICPA‐UNTDF) Ushuaia Argentina; ^7^ Department of Biology Aarhus University Aarhus C Denmark; ^8^ Facultad de Ciencias Veterinarias y Pecuarias Universidad de Chile La Pintana Santiago Chile; ^9^ Genomics in Ecology, Evolution & Conservation Lab (GEECLAB), Departamento de Zoología. Facultad de Ciencias Naturales y Oceanográficas Universidad de Concepción Concepción Chile

**Keywords:** fjords, Notothenioidei, Patagonian blennie, protandrous hermaphrodite, salinity cline, SNPs

## Abstract

Patagonia is an understudied area, especially when it comes to population genomic studies with relevance to fishery management. However, the dynamic and heterogeneous landscape in this area can harbor an important but cryptic genetic population structure. Once such information is revealed, it can be integrated into the management of infrequently investigated species. *Eleginops maclovinus* is a protandrous hermaphrodite species with economic importance for local communities that are currently managed as a single genetic unit. In this study, we sampled five locations distributed across a salinity cline from Northern Patagonia to investigate the genetic population structure of *E*. *maclovinus*. We used restriction site‐associated DNA (RAD) sequencing and outlier tests to obtain neutral and adaptive loci, using F_ST_ and GEA approaches. We identified a spatial pattern of structuration with gene flow and spatial selection by environmental association. Neutral and adaptive loci showed two and three genetic groups, respectively. The effective population sizes estimated ranged from 572 (Chepu) to 14,454 (Chaitén) and were influenced more by locality than by salinity cline. We found loci putatively associated with salinity suggesting that salinity may act as a selective driver in *E*. *maclovinus* populations. These results suggest a complex interaction between genetic drift, gene flow, and natural selection in this area. Our findings also suggest several evolutionary significant units in this area, and the information should be integrated into the management of this species. We discussed the significance of these results for fishery management and suggest future directions to improve our understanding of how *E*. *maclovinus* has adapted to the dynamic waters of Northern Patagonia.

## Graphical abstract

Patagonia is an understudied area, especially when it comes to population genomic studies with relevance to fishery management.; Using RADseq we found that neutral and adaptive loci reveal fine‐scale population structure in *Eleginops maclovinus*.; We found loci putatively associated with salinity suggesting that it may act as a selective driver in *E*. *maclovinus* populations.

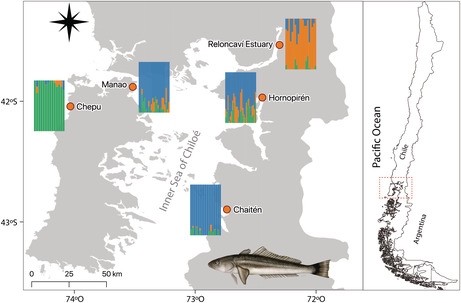

## 1 INTRODUCTION

Advances in genome sampling methods have reduced complexity (e.g., Restriction associated DNA sequencing, RADseq) and allowed the collection of an unusual amount of data to analyze the genome concerning conservation and management problems (Bernatchez et al., 2017; Xuereb et al., 2021). These data provide a great way to solve unanswered questions and have the advantage of allowing the quantification of adaptive variation unlike microsatellites (Bernatchez et al., 2017; Funk et al., 2012). Currently, researchers can differentiate neutral and adaptive variation across population information that can be incorporated into management and conservation programs to obtain better solutions on them (Xuereb et al., 2021).

Identification of evolutionary significant units (ESU) is important to guide management and conservation efforts (Funk et al., 2012) and maximize the evolutionary potential for environmental change (Bernatchez, 2016). At a fine scale, a management unit, which is included in an ESU, refers to demographically independent populations and shows significant divergence results with low gene flow (Funk et al., 2012; Moritz, 1994; von der Heyden, 2017). Finding genetic differences at a fine scale is challenging because it depends on the biological characteristics of the organisms studied (e.g., vagility) and the geomorphological conformation and environmental heterogeneity of their geographical distribution (Canales‐Aguirre et al., 2016; Jørgensen et al., 2005). For the latter, habitats such as fjords may greatly affect population genetic diversity in marine organisms due to unique environmental characteristics.

Patagonia in Chile includes a vast coastal area (240,000 km^2^; Pantoja et al., 2011). The northern region extends from latitude 41.5°S (Reloncaví Fjord) to latitude 46.5°S (San Rafael Lagune; Rodrigo, 2008) with high ecosystem productivity and heterogeneous geomorphological and physical–chemical oceanographic conditions (Pérez‐Santos et al., 2014; Ríos et al., 2016; Yevenes et al., 2017). For example, Patagonia has a saline cline pattern resulting from freshwater runoff from melting ice from the Andean Mountains (annual average caudal >300 m^3^/s) and an annual average precipitation >1000 mm (Garreaud et al., 2013). Salinity can play a role in the ontogeny resulting in a differentiated vertical distribution of eggs and yolk sac larvae (Petereit et al., 2009), and also, it influences population abundance as a result of freshwater discharges (Ojaveer & Kalejs, 2010). Also, a heterogeneous‐salinity environment can promote local adaptation in marine populations that can result in genetic population structure differences (Berg et al., 2015; Limborg et al., 2009; McCairns & Bernatchez, 2008). Such landscape characteristics influence a high diversity of marine organisms and hierarchical levels, from populations to ecosystems (Beuchel et al., 2006; Canales‐Aguirre et al., 2010, 2016; Kristoffersen & Salvanes, 2009; Olsen et al., 2002), that support and sustain economically important fisheries. Unfortunately, this area has been understudied, and no population genomics studies with relevance to fisheries management have been conducted despite the fact that this unique landscape may promote large genetic differentiation and local adaptation. Genomic studies can reveal discrete genetic groups that can be integrated into future conservation and management programs (e.g., Euclide et al., 2021; Larson, Seeb, Pascal, Templin, & Seeb, 2014; Larson et al., 2014; McKinney, Seeb, & Seeb, 2017).

*Eleginops maclovinus* (Cuvier and Valenciennes 1830) is a monotypic species and one of the few species of the Notothenioidei with a non‐Antarctic distribution (Bargelloni et al., 2000; Matschiner et al., 2015; Near, 2004). Endemic to South America, it is found close to estuaries from ~33°S on both sides of the Pacific and the Atlantic Ocean to the Beagle Channel (~54°S) (Pequeño, 1989), including in the Malvinas/Falkland Islands (Gosztonyi, 1974). This species is economically important for local communities and is caught by artisanal and recreational fishers (Gastaldi et al., 2009), with 182 tons landed in 2019 in Chile (Sernapesca, 2019). In Chilean Patagonia, there is little scientific information about this fish that can be used to identify appropriate management units. *Eleginops maclovinus* is a partial spawner with a spawning peak during late autumn in estuaries of the Chilean coast (Ruiz, 1993); it is also the most fecund of the Notothenioidei (~550,000 eggs per female, Gosztonyi, 1979). This species is a protandrous hermaphrodite, and males are often smaller in length than females (10–52 cm males and >53 cm females) (Brickle et al., 2005).

Connectivity and dispersal between populations of *E*. *maclovinus* seem to be biased by sex. Mechanical tags from females have been found up to 60 nautical miles away from their tagging location (Brickle et al., 2003), while parasites used as biological tags suggest that males are residents that do not move large distances (Brickle & MacKenzie, 2007). This information supports the idea that *E*. *maclovinus* has a structured geographic distribution; however, this has not been supported by research based on molecular approaches. For instance, phylogeographic analysis using mtDNA, which only should reflect female dispersal, showed that *E*. *maclovinus* had weak genetic differentiation, shared haplotypes among locations, and recent population expansion that would have occurred as a result of Quaternary Glaciations (Ceballos et al., 2012). In addition, a subsequent study using microsatellites revealed a low but significant level of genetic differentiation between Pacific and Atlantic populations, with Atlantic populations showing a mixed membership from the two main genetic clusters. Some degree of genetic heterogeneity was suggested within the Pacific at a lower hierarchical level (Ceballos et al., 2016). The microsatellites study also suggested that northern populations, in both Atlantic and Pacific Oceans, may harbor more genetic variability as revealed by the number of private alleles (Ceballos et al., 2016). This suggests that Northern Patagonia fjords may harbor a cryptic population genetic structure at a fine scale, as a result of the geomorphological and heterogenous landscape, which was not observed probably due to lack of sampling locations from this region or low resolution of the molecular information used.

In this study, we investigate the population genomics of *E*. *maclovinus* from Northern Patagonia, using RADseq to assess both neutral and adaptive variation. We specifically aim to (i) evaluate the extent of neutral and adaptive genetic diversity, population differentiation between locations, and estimation of effective population size (ii) correlate putative adaptive loci to environmental variables, (iii) identify putative functions for candidate loci, and (iv) discuss the implications of our results for conservation and management of the species.

## 2 MATERIALS AND METHODS

### 2.1 Sampling procedures

**FIGURE 1:**
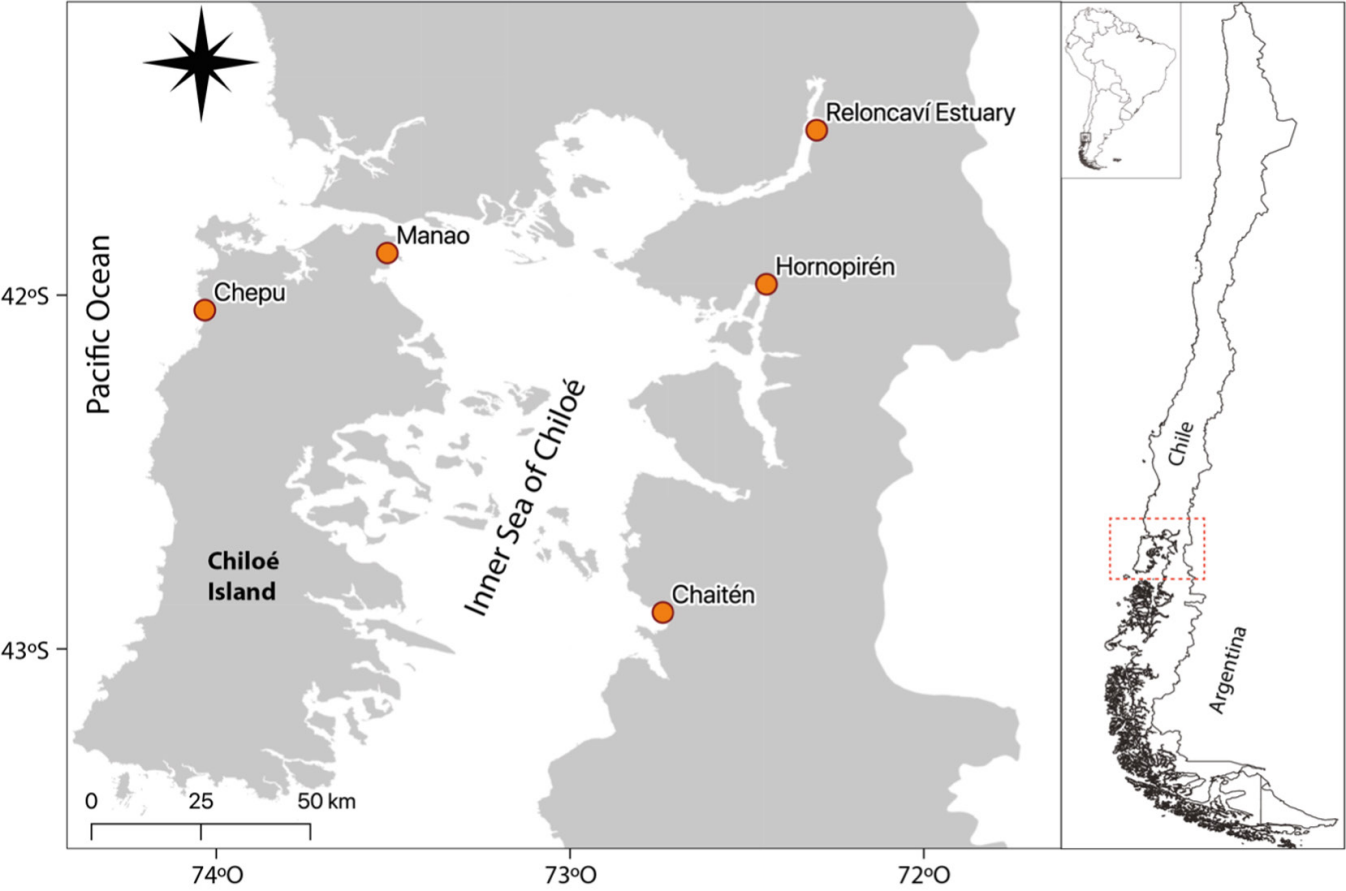
Map of sampling locations. REL, Reloncaví Estuary; MAN, Manao; HOR, Hornopirén; CHE, Chepu; CHA, Chaitén.

We collected 125 individuals using fishing nets (2”4”) or handlines from five sampling locations (25 each) between November 2018 and April 2019. Sampling locations were Reloncaví Estuary (REL), Hornopirén (HOR), Manao (MAN), Chepu (CHE), Chaitén (CHA) (Figure 1). These locations could be grouped as (i) REL (Reloncaví estuary population), (ii) MAN, HOR, CHA (Inner Sea of Chiloé populations), and (iii) CHE (oceanic population). Additionally, REL, HOR, and CHA correspond to continental locations, and MAN and CHE correspond to insular locations. For each specimen, we obtained a small piece of muscular tissue and stored it in 96% ethanol for further molecular procedures. Specimens used were collected in accordance with the national legislation of the country (Chile).

### 2.2 RADseq library and genotyping

We obtained high‐quality DNA using two steps. First, we used a traditional phenol‐chloroform DNA extraction protocol to obtain a large amount of genomic DNA; second, we purified the DNA using DNeasy Blood & Tissue Kit (Qiagen®) following the manufacturer's instructions but skipping the lysis step. We quantified the total double‐stranded DNA using a Qubit® 3.0 Fluorometer (Invitrogen) and the dsDNA BR Assay Kit, following the manufacturer's instructions. Each DNA sample was dried‐down and then normalized with sterile water to 20 ng/μl in a final volume of 10 μl (i.e., 200 ng per individual).

Restriction site‐associated DNA (RAD) libraries were prepared using the BestRAD protocol (Ackiss et al., 2020; Ali et al., 2016). Each DNA sample was digested using the SbfI‐HF® restriction enzyme (New England Biolabs) and ligated with an 8 bp unique barcode adaptor. Barcoded individual DNA samples were pooled into master libraries and fragmented to ~300–500 bp with 12–14 30 s cycles in a Q500 sonicator (Qsonica). The fragmented DNA library was bound to Dynabeads™ M‐280 Streptavidin magnetic beads (Invitrogen). Subsequently, nontarget fragments were removed by washing with a TLE buffer, and DNA was released from the beads using an incubation step. We conducted three purification steps for each library: (i) nonligated adaptors or small fragments of DNA, and enzymes for each library were removed using AMPure XP beads (Beckman Coulter); (ii) all fragments of DNA that were not barcoded were removed using Dynabeads™ M‐280 Streptavidin magnetic beads leaving only the DNA fragments with the restriction site and barcode; (iii) any residuals of small fragments of DNA and all enzymes and other impurities were also discarded using AMPure XP beads. NEBNext® Ultra™ DNA Library Prep Kit for Illumina® was used for ligation of master library barcodes, a 250‐bp insert size selection, and a 12‐cycle PCR enrichment. We confirmed enrichment and size selection by visualizing PCR products on a 2% agarose E‐Gel (Invitrogen). A final AMPure XP purification clean‐up step was conducted prior to quantifying the DNA with a Qubit® 2.0 Fluorometer. All prepared libraries for paired‐end libraries were sent to Novogene for sequencing on the Illumina NovaseqS4 platform.

### 2.3 SNP discovery

To discover and genotype SNPs from the raw RADseq data, we used a similar pipeline to the one suggested by Rochette and Catchen (2017) using the software STACKS v2.41 (Rochette et al., 2019). We selected a subset of 60 samples for testing and to select an optimal set of parameters as suggested by Paris et al. (2017). Raw sequences were demultiplexed by barcode using PROCESS_RADTAGS program, where intact barcode and cut‐site of SbfI restriction enzyme were checked, and whole reads with quality issues were discarded and trimmed to 140 bp (parameter flags: ‐e SbfI ‐c ‐q ‐r ‐t 140 ‐‐filter_illumina ‐‐bestrad). Individuals that showed <950,000 reads retained were excluded from the downstream STACKS pipeline. We kept a total of 112 individuals. Reads were then assembled to build unique stacks to identify putative loci using a maximum likelihood framework (Hohenlohe et al., 2010) with USTACKS program (parameter flags: ‐m 3 ‐M 3 ‐d –disable_gapped ‐‐model_type bounded ‐‐bound_high 0.25). A catalog of consensus loci was built with the CSTACKS program using five individuals from each sampling location that showed an amount of retained loci between average and median (parameter flags: ‐n 3 –disable_gapped). Putative loci identified for all samples were matched against the catalog with the SSTACK program (parameter flag: ‐‐disable_gapped). Data were transposed with the TSV2BAM program and built into a paired‐end contig for calling variant sites and genotyping each individual with the GSTACKS program. Finally, we used the POPULATIONS program to call genotypes in one single VCF file for the posterior filtering process. POPULATIONS program was used without any filtering at this stage to have full control of the iterative filtering process described below.

### 2.4 Bioinformatics and genotyping quality filters

We used the iterative filtering process described by McKinney et al. (2020), which applies a soft and then a more stringent threshold to remove poor‐quality loci and samples. Filtering order was: (i) minor allele frequencies, (ii) genotype rate for loci, and (iii) genotype rate for sample, after which we recalculated the proportion of missing data, and ran steps (i), (ii), and (iii) with more stringent thresholds. For minor allele frequencies (MAF), we start by removing loci with MAF ≤ 0.05, and then, MAF ≤ 0.1. For the genotype rate for loci, the first threshold was set at 25% and the second at 90%. For the genotype rate for samples, the first threshold was set at 50% and the second at 85%. Loci in Hardy–Weinberg disequilibrium (*p* < .05) were removed if they deviated in three or more populations. For tags with more than one SNP, we kept the putative SNP with the highest *F*_ST_ to reduce the influence of linked loci in the results. Additionally, paralog sequence variants were identified using HDPLOT (McKinney, Waples, et al., 2017) and then removed (parameter flags: H < 0.6; |D| < 5). Paralogs result from gene duplication events that have affected the evolution of the notothenioid genome (Chen et al., 2008, 2019). Paralogs are generally difficult to genotype reliably with RADseq data due to insufficient read depth (McKinney et al., 2018). All variants that met with these criteria were retained for further analyses (Table 1).

**TABLE 1:** Number of putative loci retained following each filtering step

Filtering steps	Sample size	Number of loci
SNP after STACKS	112	1,334,812
MAF >= 0.05^a^	112	829,446
Genotyped by locus (50%)^a^	112	168,256
Genotyped by sample (25%)^a^	109	168,256
Genotyped by locus (90%)^a^	109	38,757
Genotyped by sample (85%)^a^	101	38,757
MAF >= 0.1^a^	101	21,266
Min–mean DP 10	101	21,264
Max–mean DP 100	101	21,258
One SNP by tag	101	12,546
Hardy–Weinberg	101	12,505
Singletons (HdPlot H < 0.6; |D| < 5)	101	12,382
Neutral loci^b^	101	12,026
Adaptive loci merged^c^	101	356
Adaptive loci shared^d^	101	13

^a^ Steps included in the iterative filtering process.

^b^ The neutral dataset included all loci that were not included in the adaptive loci merged.

^c^ The adaptive loci merged dataset included all loci that were identified as an outlier in each software.

^d^ The adaptive loci shared dataset included all loci that were shared among three software (PCADAPT, FSTHET, and RDA).

### 2.5 Data analyses: Identifying neutral and adaptive loci

We used three methods to detect loci under selection (PCADAPT, FSTHET, and Redundancy Analysis). The first method inferred outliers based on principal component analysis (PCA) and was implemented using the PCADAPT package v4.3.3 (Privé et al., 2020). This method assumes that markers excessively related to population structure are candidates for local adaptation (Luu et al., 2017). The PCADAPT method uses the Mahalanobis distance (D) statistic, where a vector of the *z*‐score is derived for regressing each SNP with K principal components Luu et al. (2017). We applied Cattell's rule to choose the *K* number of the principal components (Cattell, 1966). The *p*‐values were obtained from transforming Mahalanobis distance (D) based on the chi‐square distribution (Cattell, 1966). To avoid confounding effects of the population structure, we identified an optimal *K*‐value testing from *K* = 1 to *K* = 10, and we checked the proportion of variance explained by each principal component using a scree plot using the *pcadapt* function and PCA (Figure S1). *K* = 3 was retained, and we calculated the false discovery rate of the *p*‐values associated with Mahalanobis distances using the QVALUE package (Dabney et al., 2010). A list of putative adaptive loci was obtained under an expected false discovery rate of α = 0.1. The second method corresponds to FSTHET, which identifies candidate loci by calculating smoothed quantiles between loci with strong differentiation *F*_ST_ relative to their expected heterozygosity (Flanagan & Jones, 2017). This approach does not require any assumptions about the underlying population structure and is therefore more broadly applicable than other outlier detection methods. We calculated the empirical *F*_ST_ based on Wright (1943) and expected heterozygosity. Loci were binned based on their expected heterozygosity values, sorted by *F*_ST_ value, and quantiles were calculated. Loci that showed departures from a 95% confidence interval were considered under positive or balancing selection whether they surpassed superior or inferior confidence intervals, respectively (Figure S2). Finally, we used a Genotype‐Environment Association approach (GEA) conducting a Redundancy analysis (RDA) to identify signatures of local adaptation to environmental variables. RDA is a multivariate ordination method that combines PCs from allele frequency and multivariate environmental distance matrices to produce canonical axes predicting relationships between environments and particular loci (Forester et al., 2018; Rellstab et al., 2015). We used the Bio‐Oracle dataset (Assis et al., 2018; Tyberghein et al., 2012) to obtain environmental variables for salinity, temperature, pH, oxygen, silicate, current velocity, primary production, phosphate, phytoplankton, iron, nitrate, chlorophyll a, and calcite (https://www.bio‐oracle.org/). For all these variables, we obtained values for the maximum, minimum, mean, and range of each variable when possible. RDA was performed using the VEGAN v. 2.3.4 R package (Oksanen et al., 2015). Variance inflation factor (VIF; *vif.cca* function of VEGAN) was used to ascertain the lack of multicollinearity among variables (Hair et al., 1995; Zuur et al., 2010) and excluded variables with a VIF ≥ 10 (Hair et al., 1995). Outliers were identified on each of the first three ordination axes as SNPs with a “locus score” that was ±3 SD from the mean score for that axis RDA, as suggested by Forester et al. (2018) to minimize false‐positive and false‐negative results. We then determined the correlation between each candidate SNP and one or more environmental variables.

The VennDiagram package (Chen & Boutros, 2011) was used to identify all putative adaptive loci identified and shared among the three software (PCADAPT, FSTHET, and RDA). Three datasets were built after identifying putative loci under selection: (i) neutral, (ii) adaptive loci merged, and (iii) adaptive loci shared. The neutral dataset included all loci that were not included in the adaptive loci merged (12,026 SNPs). The adaptive loci merged dataset included all loci that were identified as an outlier in each software (356 SNPs). The adaptive loci shared dataset included all loci that were shared among three software (13 SNPs). These three datasets were used for further population genomic analyses.

### 2.6 Summary statistics, population divergence, and effective population size

The summary statistics of genetic diversity expected heterozygosity (H_E_) and observed heterozygosity (H_O_) for each subset were calculated by location and conducted using the HIERFSTAT v0.04‐10 package (Goudet, 2005). The number of polymorphic loci and the effective population size (N_e_) of each location were obtained in NEESTIMATOR v2 (Do et al., 2014). The N_e_ was estimated only for the neutral dataset using the LD method (Waples, 2006) updated for missing data and following Peel et al. (2013). Values of N_e_ within corresponding 95% confidence intervals for each population were estimated using the following parameters: a minimum allele frequency cutoff of 0.01 and a random mating model.

We estimated the individual ancestry coefficients based on sparse non‐negative matrix factorization algorithms (sNMF) using the package “LEA” in R (Frichot & François, 2015). In this package, we tested each dataset to reveal the population genetic structure. We identified the best number of genetic clusters (K) based on cross‐validation and on an information theoretic measure, the cross‐entropy criterion (Alexander & Lange, 2011; Frichot & François, 2015). We iteratively tested from *K* = 1 to *K* = 10, with 10 replicates, and with 10,000 permutations per *K* using the function *obj.snmf* in LEA. We conducted the statistical procedure PCA to reduce the multivariate SNP multilocus data into two orthogonal axes using the ADEGENET v2.0 package (Jombart, 2008; Jombart & Ahmed, 2011). We used the PCA approach in all subsets obtained. We used the PCA to seek a summary of the genetic diversity among the sampled individuals ignoring the assumptions of the Hardy–Weinberg equilibrium and linkage disequilibrium, which are often required in other individual‐based models. Finally, we calculated pairwise *F*_ST_ values for populations and performed significance tests for pairwise using 10,000 permutations in the STAMPP package (Pembleton et al., 2013). The *F*_ST_ estimation was following the Wright (1949) method but was corrected by the unequal population size as updated by Weir and Cockerham (1984) (see Pembleton et al., 2013). This analysis was completed for each dataset. To understand the process that led to the population structure, we calculated directional migration rates among locations using the *divMigrate* function (Sundqvist et al., 2016) included in the diveRsity package (Keenan et al., 2013). Using all datasets, we explored the migration rates using the effective number of migrants index (Nm; Alcala et al., 2014), as a statistic to calculate relative migration. We used 1000 bootstraps to test the statistical significance of directional migration, and no filter threshold was applied to see all migration rates estimated. The conception behind the *divMigrate* function is that for each pair of populations, a hypothetical pool of migrants is created using the allelic frequencies inferred from the two populations compared. Then, a measure of genetic differentiation is estimated for the hypothetical pool and between each pair of population. This directional genetic differentiation obtained is then used to calculate the relative migration between the two populations. Additionally, a one‐way anova was performed to evaluate whether the Nm was different for the three different datasets. This analysis was conducted using the R package rstatix v0.6.

### 2.7 Putative function from Blast2Go

We conducted loci annotation to identify a putative function for candidate variants underlying positive selection obtained from PCADAPT, FSTHET, and RDA. We used the software Blast2Go included in OmicsBox following the annotation pipeline described by Götz et al. (2008). Briefly, we compared our candidate loci against the NCBI genomic database translating the sequences from nucleotide to protein using BLASTX. Then, we mapped homologous sequences to Gene Ontology (GO) terms. Finally, sequences were annotated by applying the Blast2GO annotation rule (see Götz et al., 2008). We tabulated the Tag_SNP from our read; a method that identified the outlier, environmental variable associated, Gene name, Gene Ontology ID, and their respective GO names.

## 3 RESULTS

### 3.1 Sequencing, genotyping quality filters, and datasets

**FIGURE 2:**
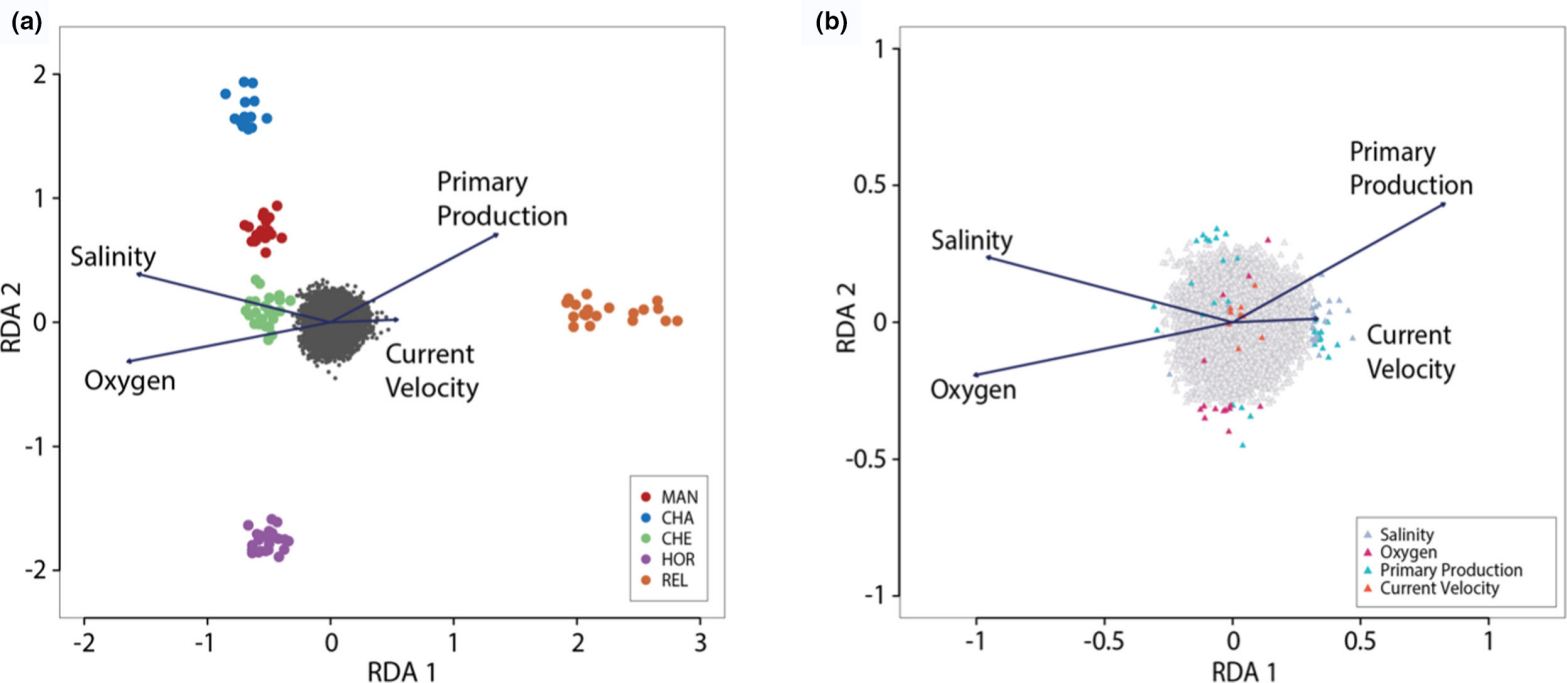
Triplots for RDA axes 1 and 2 for (a) individuals in sampling locations and (b) SNPs. In panel a, loci are represented by the dark gray cloud of points. Dots correspond to individuals, which are colored by sampling location. Arrows (vectors) correspond to environmental predictors. In panel b, colored triangles represent loci associated with the environmental predictors.

After excluding individuals with low number of reads retained, we obtained RAD data from 112 individuals that ranged from 971,305 to 14,872,661 reads with a median of 5,821,883 reads. The STACKS pipeline without filter revealed a total of 1,334,812 (242,278 RADtag) putative SNPs. 98.41% (1,313,546) of SNPs were removed by the iterative quality filters (MAF and missing data), increasing to 99.06% removed by the read‐depth filter (8); keeping only one SNP per tag (8712), and finally, the percentage increased to 99.07% after removing Hardy–Weinberg disequilibrium departure loci (41) and paralogs (123). The whole filtering process resulted in a final dataset of 101 individuals and 12,382 high‐quality SNPs. Outlier tests using PCADAPT identified 26 putative adaptive (0.2%), while FSTHET revealed 332 loci for positive selection (3%). The global model of the multilocus genotype‐environment RDA (Figure 2a) conducted using all loci was significant (anova *F*_1,21_; *p* = .001) with the first three components explained 26.87%, 24.76%, and 24.60% of the variation for RDA1 to RDA3, respectively. We excluded the variables calcite, chlorophyll a, nitrate, iron, phosphate, phytoplankton, silicate, temperature, and pH that presented a VIF ≥ 10, and we kept only maximum dissolved molecular oxygen (VIF: 6.173), mean of primary productivity (VIF: 7.985), mean salinity (VIF: 4.580), and minimum current velocity (VIF: 3.611). For these variables a total of 78 putative adaptive loci were found, where 25 loci were correlated to oxygen, 14 loci to primary productivity, 30 loci to salinity, and 9 loci to current velocity (Figure 2b). Finally, a total of 356 adaptive loci were identified among PCADAPT, FSTHET, and RDA, while13 loci were shared among the three software. Four loci were unique for PCADAPT, 267 for FSTHET, and 18 for RDA (Figure S3).

### 3.2 Summary statistics, population divergence, and effective population size

Summary statistics of genetic diversity revealed similar values for each location within each dataset (Table 2). For the neutral dataset, H_O_ ranged from 0.301 to 0.314, and H_E_ from 0.317 to 0.324. For the adaptive loci merged dataset, H_O_ ranged from 0.258 to 0.299 and H_E_ from 0.286 to 0.322. For the adaptive loci shared dataset, H_O_ ranged from 0.151 to 0.371 and H_E_ from 0.161 to 0.436. The number of polymorphic loci for the neutral dataset was 12,021 for REL, 12,021 for MAN, 12,025 for HOR, 11,995 for CHE, and 12,000 for CHA (Table 2). Most estimates of effective population size for the neutral dataset were finite and varied by two or three orders of magnitude across locations; the N_e_ estimate ranged from 570.3 in Chepu to 16,238.7 in Chaitén (Table 2). Only the Chaitén confidence interval included an infinite value.

**TABLE 2:** Summary statistics of populations analyzed, observed heterozygosity (H_O_), expected heterozygosity (H_E_), percentage of polymorphic loci (PL), and effective population size (N_e_).

Location	Neutral						Adaptive loci merged		Adaptive loci shared	
	*N*	H_O_	H_E_	% PL	N_e_ ^a^	CI	H_O_	H_E_	H_O_	H_E_
REL	20	0.313	0.324	99.7	1296.6	1190.7–1423.2	0.299	0.322	0.371	0.436
MAN	23	0.307	0.324	99.8	3543.8	2941.3–4455.4	0.276	0.302	0.191	0.215
HOR	27	0.301	0.317	100	2946.1	2569.4–3452	0.277	0.300	0.164	0.161
CHE	14	0.314	0.321	98.5	570.3	536.4–608.6	0.274	0.294	0.151	0.172
CHA	17	0.303	0.319	99.3	16238.7	6780.8–Inf	0.258	0.286	0.193	0.191

*Note*: Index was calculated using 12,026 SNPs for neutral loci, 356 SNPs for merged adaptive loci, and 13 SNPs for shared adaptive loci datasets.

Abbreviations: CHE, Chepu; CHA, Chaitén; HOR, Hornopirén; Inf, infinite; MAN, Manao; REL, Reloncaví Estuary.

^a^ Effective population size estimated based on linkage disequilibrium (LD; Waples & Do, 2010) and only for neutral dataset.

**FIGURE 3:**
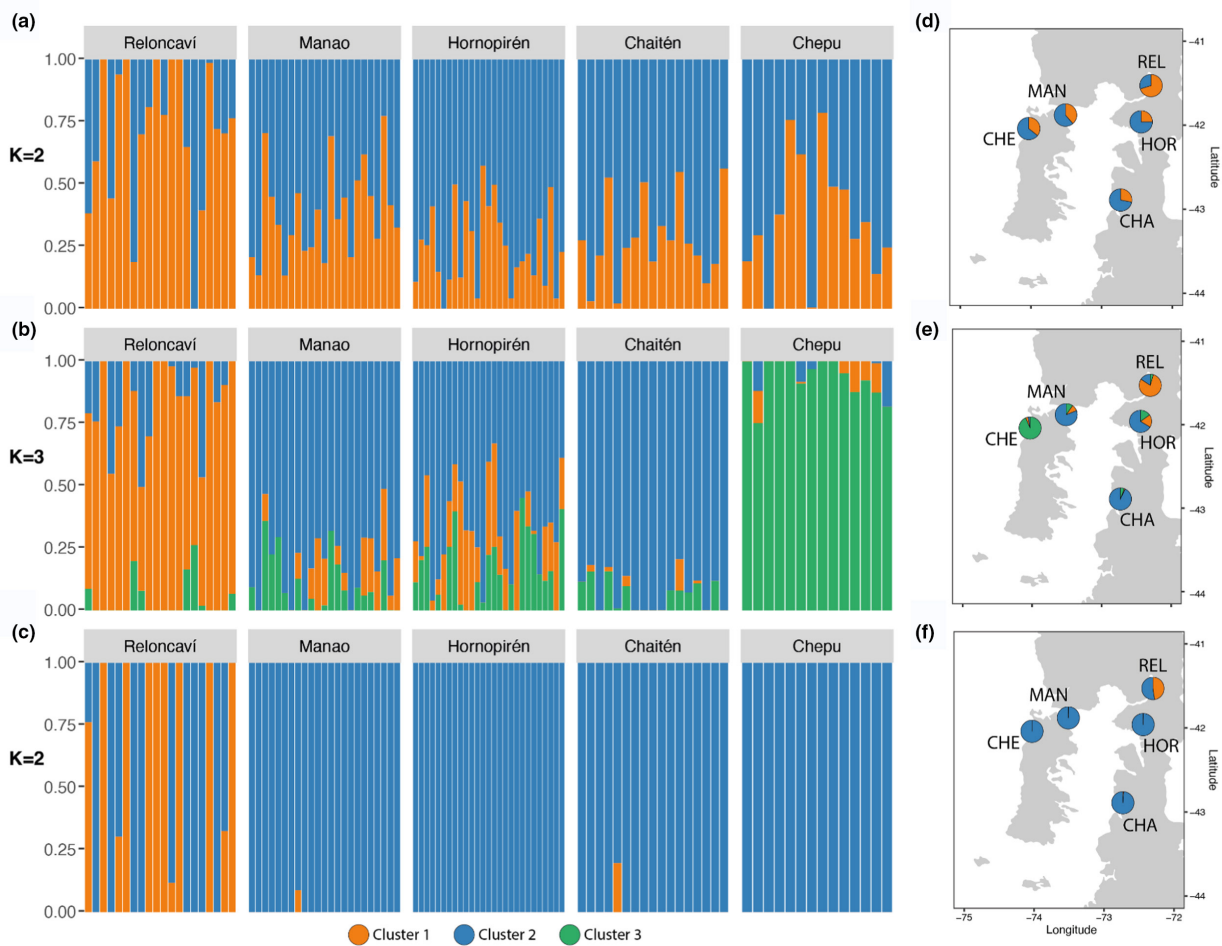
Admixture results showing the estimated population admixture coefficients (Q) for each individual, whose genome is broken into colored segments representing the proportion of that individual's genome derived from each of the K inferred clusters. (a) Neutral dataset (12,026 SNPs), (b) adaptive merged loci (356 SNPs), and (c) adaptive shared loci (13 SNPs). Mean admixture proportions by location in (d) neutral loci, (e) adaptive merged loci, and (f) adaptive shared loci.

The lowest value of the cross‐entropy suggested *K* = 1 for neutral, *K* = 3 for adaptive merged, and *K* = 6 for adaptive shared dataset (Figures S4–S6). We surveyed the admixture result from *K* = 2 to *K* = 5 and decided to keep *K* = 2 for neutral, *K* = 3 for adaptive merged, and *K* = 2 for the adaptive shared dataset (Figure 3a–c) because of the shared similarity with PCA (result below). For the neutral dataset (*K* = 2), the first group included REL (~0.70 of admixture), and the second group included MAN, CHE, HOR, and CHA (admixture ranging between ~0.7 and ~0.75) (Figure 3d). For the adaptive merged dataset (*K* = 3), the first group included REL (~0.75 of admixture), the second included MAN, HOR, and CHA (admixture ranging between ~0.7 and ~0.95), and the third group included CHE (~0.95 of admixture) (Figure 3e). For *K* = 2 in the adaptive shared dataset, the first group included REL (~0.55 of admixture) and the second one included MAN, CHE, HOR, and CHA (admixture ranging between ~0.65 and 1), (Figure 3f). The Reloncaví Estuary location was clearly different in admixture analyses for all datasets (Figure 3). Mean admixture proportions by location in putative neutral, adaptive merged, and adaptive shared loci are represented in Figures S7–S9.

**FIGURE 4:**
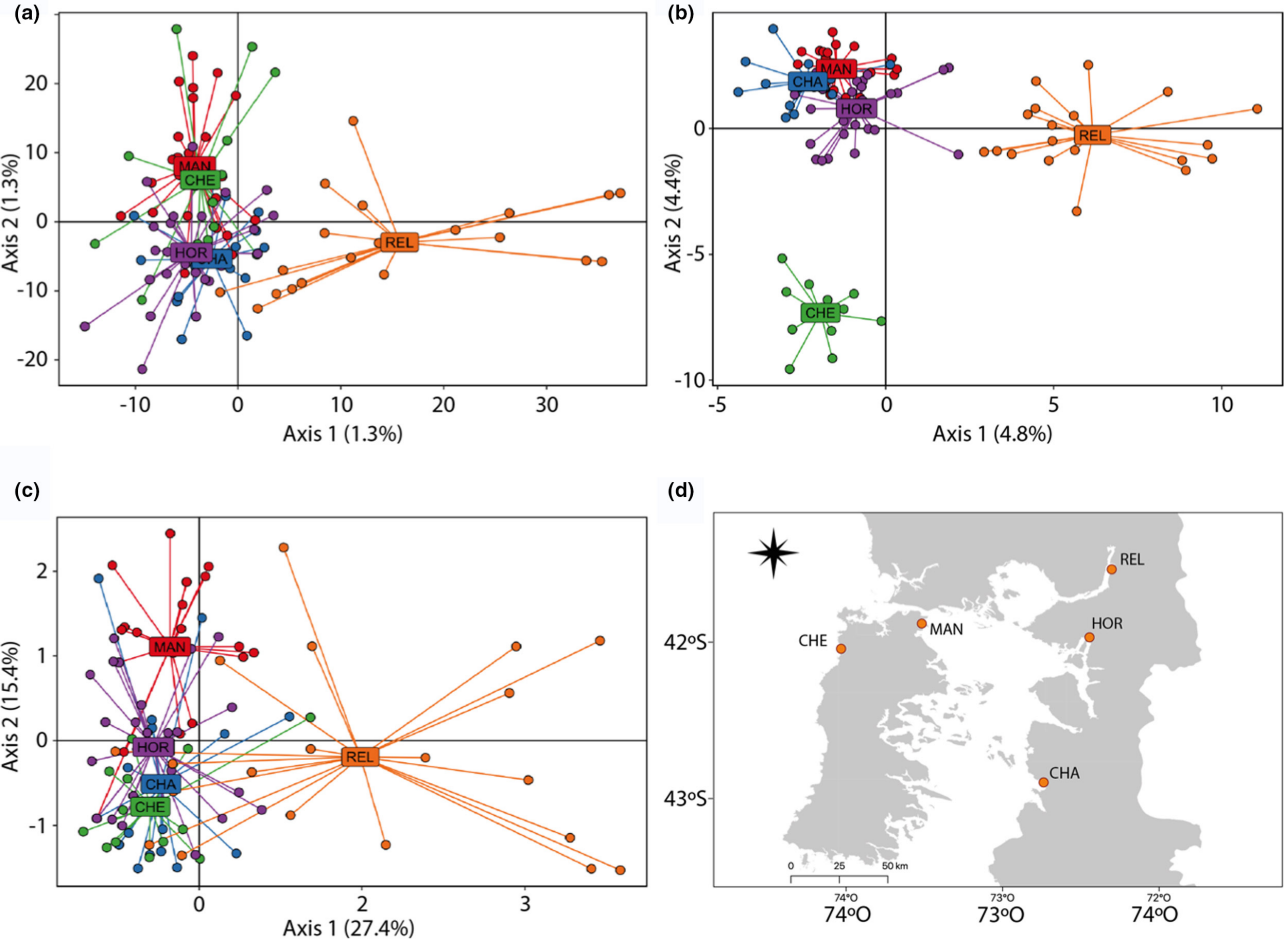
Individual‐based principal component analysis for north Patagonian populations using (a) neutral dataset (12,026 SNPs), (b) adaptive merged loci (356 SNPs), and (c) adaptive shared loci for PCADAPT, FSTHET, and RDA analyses (13 SNPs). (d) Map indicating locations for spatial context; REL, Reloncaví Estuary; MAN, Manao; HOR, Hornopirén; CHE, Chepu; CHA, Chaitén.

Based on PCA, we observed slight differences in structuration patterns among the three datasets. The neutral loci dataset was only able to show clear differences between two groups (Figure 4a) while the adaptive merged loci dataset identified three distinct groups (Figure 4b), (i) REL, (ii) MAN, HOR, CHA, and (iii) CHE. The use of adaptive merged loci increased the variance explained by PCs (4.8% and 4.4% for PC1 and PC2, respectively) when compared to the neutral dataset (1.3% for PC1 and PC2), and decreased dispersion of individuals within groups. Contrarily, the shared adaptive loci dataset did not show the same pattern for CHE when compared to the merged adaptive loci dataset. In this PCA, which showed the highest amount of variation explained by PCs (27.4% and15.4% for PC1 and PC2, respectively), CHE seems to be closer to CHA and HOR but slightly differentiated from MAN (Figure 4c). The REL population was identified as a different genetic group than other populations in all analyses across the three datasets.

**FIGURE 5:**
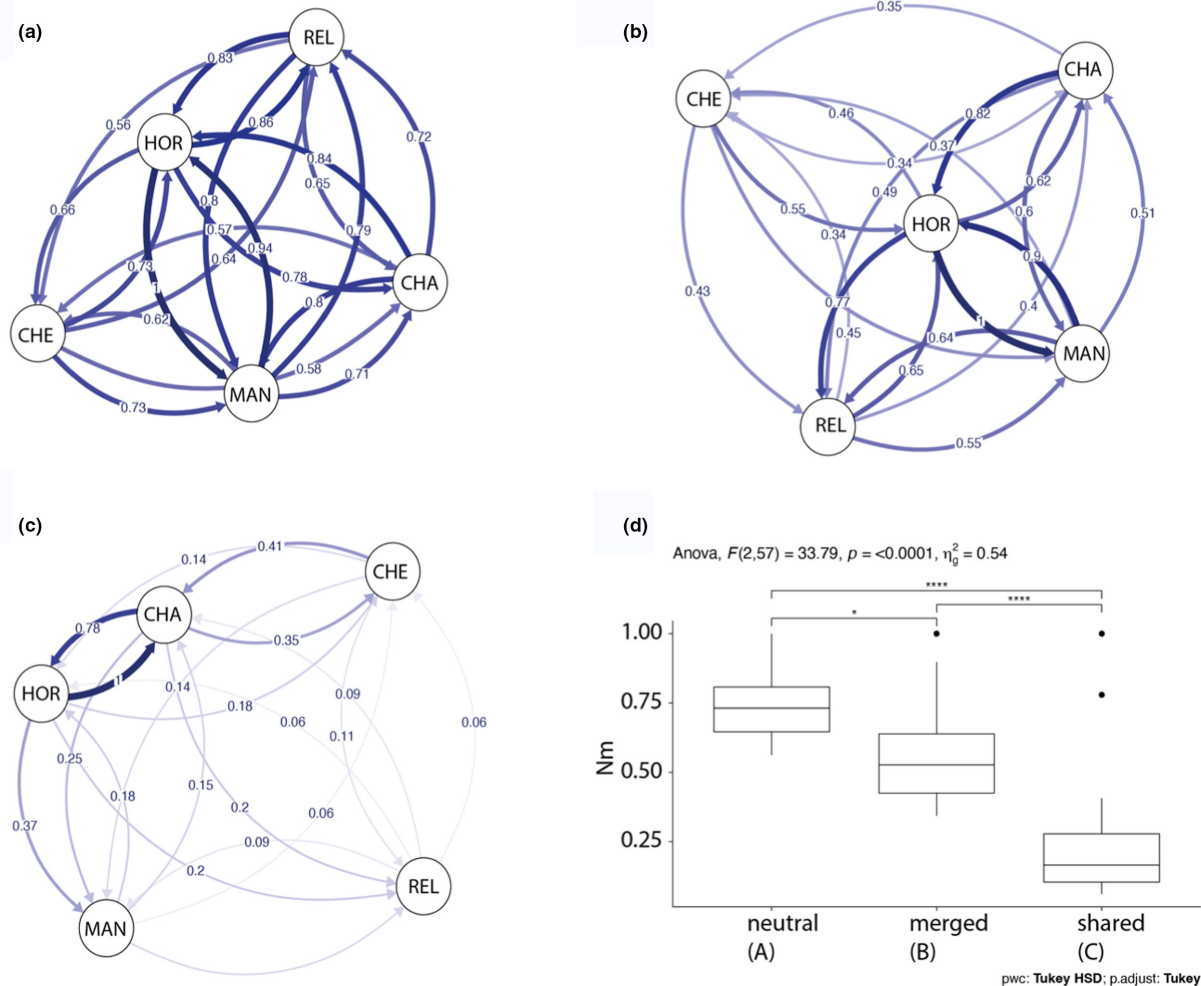
Directional relative migration network including all relative migration values among locations by (a) neutral loci, (b) adaptive merged loci, and (c) adaptive shared loci. Box plots (d) for one‐way anova comparing differences among Nm distance and the three different datasets. *p < .05; ****p < .0001. REL, Reloncaví Estuary; MAN, Manao; HOR, Hornopirén; CHE, Chepu; CHA, Chaitén.

Pairwise *F*_ST_ values, using Wright (1949) method, for the neutral dataset ranged from 0.003 for the MAN‐HOR, MAN‐CHA, and HOR‐CHA comparisons with 0.006 for the CHE‐REL comparison (Table 3). For the merged adaptive loci dataset, pairwise *F*_ST_ values ranged from 0.043 for MAN‐HOR to 0.114 for the CHE‐CHA comparison; while the shared adaptive loci dataset values ranged from 0.001 for HOR‐CHA to 0.194 for REL‐HOR (Table 3). Overall, relative migration rates using neutral dataset (Figure 5a) showed high gene flow between locations, with rates ranging from 0.563 to 1 (median = 0.731; SD = 0.121). For merged adaptive loci (Figure 5b), the relative migration rates ranged from 0.343 to 1 (median = 0.527; SD = 0.190) while shared adaptive loci (Figure 5c) ranged from 0.062 to 1 (median = 0.166; SD = 0.243). In all datasets, the HOR location was involved in a maximum migration rate estimated (Neutral MAN→HOR, Merged HOR → MAN, shared HOR → CHA). The one‐way anova showed significant differences for all Nm databases (*F*_2,57_; *p* < .0001), where neutral loci showed a higher relative migration rate comparing with adaptive loci (Figure 5d).

**TABLE 3:** Pairwise *F*_ST_ values for neutral and adaptive datasets

	Neutral					Adaptive loci merged					Adaptive loci shared				
	REL	MAN	HOR	CHE	CHA	REL	MAN	HOR	CHE	CHA	REL	MAN	HOR	CHE	CHA
REL															
MAN	0.005					0.076					0.175				
HOR	0.005	0.003				0.061	0.043				0.194	0.075			
CHE	0.006	0.004	0.005			0.107	0.102	0.082			0.187	0.185	0.090		
CHA	0.005	0.003	0.003	0.005		0.094	0.073	0.055	0.114		0.150	0.100	0.001	0.024	

*Note*: Pairwise Fst index was calculated using 12,026 SNPs for neutral loci, 356 SNPs for merged adaptive loci, and 13 SNPs for shared adaptive loci. *F*_ST_ estimation was using Wright (1949) methods but corrected by Weir and Cockerham (1984) for uneven population size (see Pembleton et al., 2013).

Abbreviations: CHE, Chepu; CHA, Chaitén; HOR, Hornopirén; MAN, Manao; REL, Reloncaví Estuary.

### 3.3 Putative function from Blast2GO

From 356 putative candidate loci (FSTHET, PCADAPT, and RDA), 124 loci were blasted in BlastX, 111 loci mapped were of homologue sequences to GO terms, and finally, 98 loci were annotated to GO terms. Based on these results, we found a variety of candidate genes whose functions involved mitotic cytokinesis, epithelial cell differentiation, embryonic morphogenesis, chondrocyte differentiation, and kidney development, among others (Table S1).

## 4 DISCUSSION

Under the assumption that heterogeneous landscape, low vagility, and biological conditions can result in population divergence in *Eleginops maclovinus*, we aimed to (i) disentangle the differences in neutral and adaptive genetic variation, (ii) correlate putative adaptive loci to environmental variables, and (iii) identify putative functions for candidate loci. PCA and membership analyses revealed two (neutral loci) and three (adaptive loci) clusters, none of them previously described for this species. Neutral loci suggest a spatial pattern of structuration with gene flow, while adaptive loci suggest spatial selection by environmental association. We identify candidate loci for divergent selection mainly associated with biological processes (e.g., DNA repair, sodium ion transmembrane transport, and metabolism). Contrasting N_e_ estimations among populations were found, showing Chepu had the lowest N_e_ estimated, nonetheless, this population still presents a high gene flow among other populations. Overall, our results uncover a hidden fine‐scale population structure and adaptation despite considerable gene flow in *E*. *maclovinus* along with its North Patagonian distribution (i.e., Reloncaví Estuary). Identification of these groups will facilitate the development of conservation and management measures for this species.

### 4.1 Neutral genetic variation

We found fine‐scale genetic spatial pattern of structuration with gene flow based on neutral genomic data. We identified two genetic groups along the main PCA axis; the strongest genetic differentiation occurred between Reloncaví Estuary and all other populations. Studies in *E*. *maclovinus* using mtDNA and microsatellites showed contrasting results. For example, based on the Cyt‐b fragment, Ceballos et al. (2012) showed low genetic differentiation between five populations of *E*. *maclovinus* located between Pacific and Atlantic distribution. They suggested current and historical connectivity between populations from their expansion from the middle Pleistocene. Later, using microsatellites, low but significant regional differentiation between the Pacific Ocean and Atlantic Ocean locations was found (Ceballos et al., 2016). Similar studies using microsatellites did not find population structure in the Patagonian area from the Pacific Ocean (Canales‐Aguirre et al., 2010, 2018). Our findings differ from at scale level with previous results and reinforce that genotyping tools such as RADseq increase the power to resolve shallow population structure in fish with gene flow (Hollenbeck et al., 2019; Larson, Seeb, Everett, et al., 2014; Luikart et al., 2003) when other lower resolution genomic approaches cannot.

Dispersal and reproductive attributes could contribute to the observed population pattern. Theoretically, hermaphroditic species tend to have more structured populations than gonochoristic species (Chopelet et al., 2009; Coscia et al., 2016), but information supporting this hypothesis is scarce. For example, Chopelet et al. (2009) conducted a meta‐analysis testing this hypothesis and found no supporting evidence. They suggested that dispersal capacities and environmental barriers can play an underlying role in the variance of the genetic structuring of marine fish populations. Dispersal behavior in *E*. *maclovinus* has been recorded using biological (parasites) and mechanical tags (Brickle et al., 2003; Brickle & MacKenzie, 2007). Both studies indicate that juveniles (mainly males) tend to be residents and larger fish (mainly female) can migrate, comparatively, large distances (up to 60 nautical miles, i.e., 111 km) (Brickle et al., 2003; Brickle & MacKenzie, 2007). In our case, the maximum geographic distance between sampling locations is approximately 230 km (REL‐CHE). It seems large enough to avoid connectivity but that is in a scenario where sampling locations are unique populations in the sampling area where *E*. *maclovinus* inhabits, which is not true. In our fine‐scale sampling area, there are other estuaries between our sampling locations where we can find individuals of *E*. *maclovinus*. Individuals could move among locations in a stepping stone fashion differentiating extreme locations. This can also bring to mind isolation by distance pattern, that is reinforced by our mantel analyses tested (Figure S12). The relative migration rates estimated show high connectivity among locations (median = 0.73), suggesting that individuals disperse freely in this area, even when location REL is more isolated as suggested PCA and admixture analyses. Passive dispersal by early stages (egg and larvae) is uncertain because there is no information on whether *E*. *maclovinus* have pelagic or benthic eggs or whether spawning occurs in open sea and in estuaries. Hence, it is hard to suggest how drifting of early stages can connect populations through gene flow as has been suggested for several marine species (Benestan et al., 2021). Overall, this result indicates that the life‐history trait is not enough to promote population structure in *E*. *maclovinus*, but this is maintained by a complex interaction between migration and selection (see Adaptive variation section).

The geomorphology of the Northern Patagonia and the pattern of sea currents provide further evidence for the differences observed. The presence of channels, estuaries, close sounds, and fjords can be an efficient barrier for dispersal at different life‐history stages. For example, it has been suggested that genetic isolation is related to the shallow sill depth and life‐history behavior in *Benthosema glaciale* (Kristoffersen & Salvanes, 2009). The Reloncaví Estuary is 55 km long, with geomorphological and environmental characteristics that make this a unique area (Castillo et al., 2016, 2017). For instance, superficial currents resulting from wind, flows‐down to the Reloncaví Sound in winter and flows‐up in spring and summer (Castillo et al., 2016, 2017). The latter could promote a safe environment for the early stages of marine organisms. Our samples were collected in the head of the estuary, a sheltered area, less influenced by sea tidal change and more by freshwater runoff. The sea current in Reloncaví Estuary mainly goes from the head to the mouth of the estuary (Castillo et al., 2016, 2017); however, it seems that some individuals are more similar to Hornopirén and Chaitén suggesting dispersal based on PCA. This is supported by the admixture of some individuals in the membership analyses and the cluster distribution by location. This singular geomorphology could promote a similar pattern of genetic diversity for other species. Unfortunately, to date, there are no genetic studies in fish or other marine taxa using genomic analyses for this specific microgeographical area.

### 4.2 Adaptive variation

We identified two and three groups of samples in the shared and merged adaptive datasets, respectively. The 13 loci for the shared and 356 loci for the merged adaptive datasets give some clues about their effect on structuration patterns. We suspected that a small amount of adaptive SNPs could make a difference in the structuration pattern. These SNPs showed only a small fraction of the genome, leaving genomic regions not explored that may have likely loci under selective pressures. These 13 loci only reflect adaptive differences found for REL but not for CHE such as merged adaptive dataset shown. The underlying idea to highlight the results of both, shared and merged adaptive datasets was to be aware of outcomes minimizing the type I error and type II error, respectively; thus, do not miss loci under weak selection (Hoban et al., 2016; Lotterhos & Whitlock, 2015). Different statistical approaches to find outlier loci can identify different loci under selection (Lotterhos & Whitlock, 2015). What approach is better will depend on the demographic history of the population surveyed (Lotterhos & Whitlock, 2015). In our case, we do not know the real demographic history of *E*. *maclovinus*, therefore using all approaches and including all putative adaptive loci give us a broad view of how selective pressure is acting. Both adaptive datasets reinforce the idea that REL is different from the rest of the locations, but only the merged adaptive loci permit us to separate also CHE. This latter pattern fit well with the different seascape in the studied area (i.e., REL, influenced by runoff of fresh water; MAN‐HOR‐CHA, influenced by a mix of runoff fresh and marine waters; CHE, influenced by marine water).

The GEA analysis provided evidence for local adaptation to current velocity, primary productivity, oxygen, and salinity; regardless of the database used. Those environmental variables seem to be exerting selective pressures on *E*. *maclovinus*. A major effect of these variables is expected in the early development stages, where survival to recruitment is crucial. For instance, recruitment variability is explained by environmental stability and food availability (Houde, 2008). A low sea current velocity provides a stable environment, while primary productivity is an indirect proxy for food availability (Gove et al., 2016; Fox et al., 2018). Oxygen plays a role in energetic metabolisms and neuromuscular processes, which are important during embryonic development and environmental stress (Epelboin et al., 2016; Moreira et al., 2018). In addition, salinity modifies the egg and larvae distribution (Petereit et al., 2009) and can provide a challenging environment for adaptation in fish. To date, there is scarce information about how these variables specifically impact the ontogeny of *E*. *maclovinus*. For instance, juveniles of *E*. *maclovinus* have a faster growth rate in intermediate salinity environments (iso‐osmotic and hyperosmotic; Vargas‐Chacoff et al., 2015) and with food availability conditions (Vanella et al., 2017). Oxygen consumption as a proxy of metabolisms showed minor changes comparing gill and kidney tissues with liver tissue, suggesting that *E*. *maclovinus* can adapt to different salinities (Vargas‐Chacoff et al., 2014). Although we did not find the association with temperature, studies have shown that *E*. *maclovinus* could reorganize components for the intermediary metabolism to respond to climate change (Oyarzún et al., 2017). Additionally, it has been proposed a trade‐off between growth and swimming activity to allocate energy, where juveniles allocate more energy to swimming in low temperatures than physiological functions like growth (Vanella et al., 2012).

All genetic groups identified with the adaptive datasets are distributed in an environmental cline and some locations are more influenced by marine conditions than others. Divergence in adaptive loci reinforces the idea that such markers may experience selection by environmental pressures (Hollenbeck et al., 2019). Similar studies outside of this geographical area have been conducted. For instance, in the estuarine fish *Sciaenops ocellatus* from the Atlantic Ocean in the United States and Mexico, neutral and adaptive loci show a similar pattern for population structure, but discordance in the *F*_ST_ magnitude for outlier loci was greater than in neutral loci (Hollenbeck et al., 2019). Differences in habitat from dissolved inorganic phosphates, average wind speed, and minimum ocean salinity can play a role in adaptive divergence. Environmental patterns in the Reloncaví Estuary indicate a higher influx of freshwater than Chepu, hence salinity may be a driver for this divergence. Additionally, winds in the Reloncaví Estuary result in a more protected area compared with the Chepu. Araneda et al. (2016) identified divergent adaptive loci for *Mytilus chilensis*, a native mollusk species, in the same area where samples were collected for this study. They found that factors such as salinity, water discharge by rivers, glacial melt, and precipitation cause differences in the habitat between the Reloncaví Estuary and the exposed area in Chiloe (Araneda et al., 2016). Two main genes identified in our study, SNX19 and DYNC2H are candidates for salinity adaptation. The SNX19 participates in regulating vesicle trafficking, which can confer salt tolerance and the DYNC2H1, which has been associated with “renal water homeostasis,” “vasopressin‐regulated water reabsorption,” and “urea transport” (Zhou et al., 2018). Other putative candidate loci annotated as GO terms in Blast2GO genes that may be interesting for *E*. *maclovinus* are disintegrin and metalloproteinase with thrombospondin motifs 1 (ADAMTS1), palladin isoform X2 (PALLD) Glutaminase kidney isoform (GLS), Rock1 kinase (ROCK1), Elongation factor‐like GTPase 1 (EFL1), collagen alpha‐1 (IV) chain (COL4A1), and sorting nexin‐33 (SNX33). Further, our findings reinforce the idea that salinity levels can act as a selective force in *E*. *maclovinus* populations. These results open new questions to investigate in *E*. *maclovinus* given its euryhaline and eurythermic conditions. For instance, are polygenic selection driving adaptation in *E*. *maclovinus* populations? Is the architecture genetic of adaptive traits ruled by polygenic with either large or small variance in allele effect size? Further studies should be conducted to a better understanding of adaptation in *E*. *maclovinus*.

### 4.3 Management implications

Our results provide the first report of the fine‐scale spatial pattern of structuration with gene flow and spatial selection by the environmental association in *E*. *maclovinus*. This information can be used to improve the currently weak management measures that only cover regulations for the type of fishing gear used. The economic importance and conservation status of *E*. *maclovinus* populations make the lack of regulatory measures for fishery management an important issue. From an economic perspective, *E*. *maclovinus* is important for the activity of local artisanal fisherman and recreational anglers (Sernapesca, 2019). Thus, our findings support the presence of different conservation/management units at a fine scale.

Stock assessment for hermaphroditic marine species is a challenge for fishery managers due to their sex ratio bias. In protandrous species, the sex ratio is skewed to males (Allsop & West, 2004) and every year fisheries often remove larger/older individuals (females) from populations, which can result in evolutionary changes in exploited populations. First, a decrease in population fitness or change of size for changing sex. Fecundity as a fitness trait in females increases with age and size; if older females are removed from the population the fitness of the population will decrease. This increases the risk of populations that show a small effective population size, such as Chepu (572) or the Reloncavi Estuary (1309). Second, Allsop and West (2003) found that hermaphrodite fish change their sex when they reach 80% of their maximum size and are 2.5 times their age of maturity. Nonetheless, the length for sex change can be strongly modified by fisheries. For instance, *Semicossyphus pulcher* found that males and females mature early in locations with intensified recreational or commercial fisheries (Hamilton et al., 2007). For *E*. *maclovinus* information about maturity is scarce. Brickle et al. (2005) indicate that males mature around 30.73 cm and females between 67 and 78 cm LT (maturation stage III). Currently, there is no information about maturity in both males and females in the areas sampled only for small‐scale commercial fisheries. Therefore, the effect of removing larger individuals could bring evolutionary changes in a population of *E*. *maclovinus*, impacting their conservation and management.

### 4.4 Future directions

We showed that using relatively dense genomic information allows us to refine the population structure pattern in *E*. *maclovinus* in a particular area in its distributional range, whereas previous studies indicated only weak genetic differentiation in a large geographical area (Ceballos et al., 2012, 2016). Future research should include the whole distribution of the species; making it possible to assess more populations along and out of the heterogeneous landscape in Patagonia and identify whether environmental variables are associated with the current diversity and population structure. Additionally, other types of genomic variation should be examined such as copy number of variations or structural variants (e.g., Barth et al., 2019; Cayuela et al., 2021), which are also informative for identifying structuration patterns (Mérot et al., 2020). Knowing the polygenic architecture associated with adaptation to environmental variables ‐combining approaches of population genomics and quantitative genetics (Gagnaire & Gaggiotti, 2016) would allow a better understanding of how the population of *E*. *maclovinus* adapts to its habitats (see examples in other taxa, Babin et al., 2017; Bernatchez et al., 2019; Xuereb et al., 2018). Moreover, given the adaptive variation found, further studies should focus on identifying environmental selective pressure associated with phenotypical and ecological traits. Finally, this study opens a new path for research in *E*. *maclovinus*, that can be started in the near future, using long‐term monitoring of genetic diversity.

## AUTHOR CONTRIBUTIONS

**Cristian B. Canales‐Aguirre:** Conceptualization (lead); data curation (equal); formal analysis (equal); funding acquisition (lead); investigation (lead); methodology (equal); project administration (lead); software (equal); validation (equal); visualization (equal); writing – original draft (equal); writing – review and editing (equal). **Wes Larson:** Methodology (equal); writing – original draft (equal); writing – review and editing (equal). **Garrett McKinney:** Methodology (equal); software (equal); writing – original draft (equal); writing – review and editing (equal). **C. Eliza Claure:** Data curation (supporting); formal analysis (supporting); software (supporting); visualization (equal); writing – original draft (equal). **J. Dellis Rocha:** Data curation (supporting); formal analysis (supporting); visualization (supporting); writing – original draft (equal). **Santiago Ceballos:** Writing – original draft (equal); writing – review and editing (equal). **Maria Ignacia Cadiz:** Methodology (supporting); writing – original draft (supporting); writing – review and editing (supporting). **Jose Yanez:** Investigation (supporting); writing – original draft (equal); writing – review and editing (equal). **Daniel Gomez‐Uchida:** Writing – original draft (equal); writing – review and editing (equal).

## FUNDING INFORMATION

This research and the APC was funded by ANID/CONICYT FONDECYT Iniciación no. 11180897.

## CONFLICT OF INTEREST

All authors claim no conflict of interest.

## Supporting information


Appendix S1

## Data Availability

All raw data analyzed for this manuscript have been uploaded to NCBI's SRA database (BioProject ID PRJNA883977). Genotypes are archived on DRYAD (https://doi.org/10.5061/dryad.mkkwh711v).
