# Supplementary material for: Neutral and adaptive loci reveal fine‐scale population structure in Eleginops maclovinus from north Patagonia
Source: Ecol Evol. 2022 Oct 3;12(10):e9343. doi: 10.1002/ece3.9343 (PMC9530513; doi:10.1002/ece3.9343)
Supplement: Supplementary file 1 — Appendix S1 [file ECE3-12-e9343-s001.docx]

**Supplementary data**


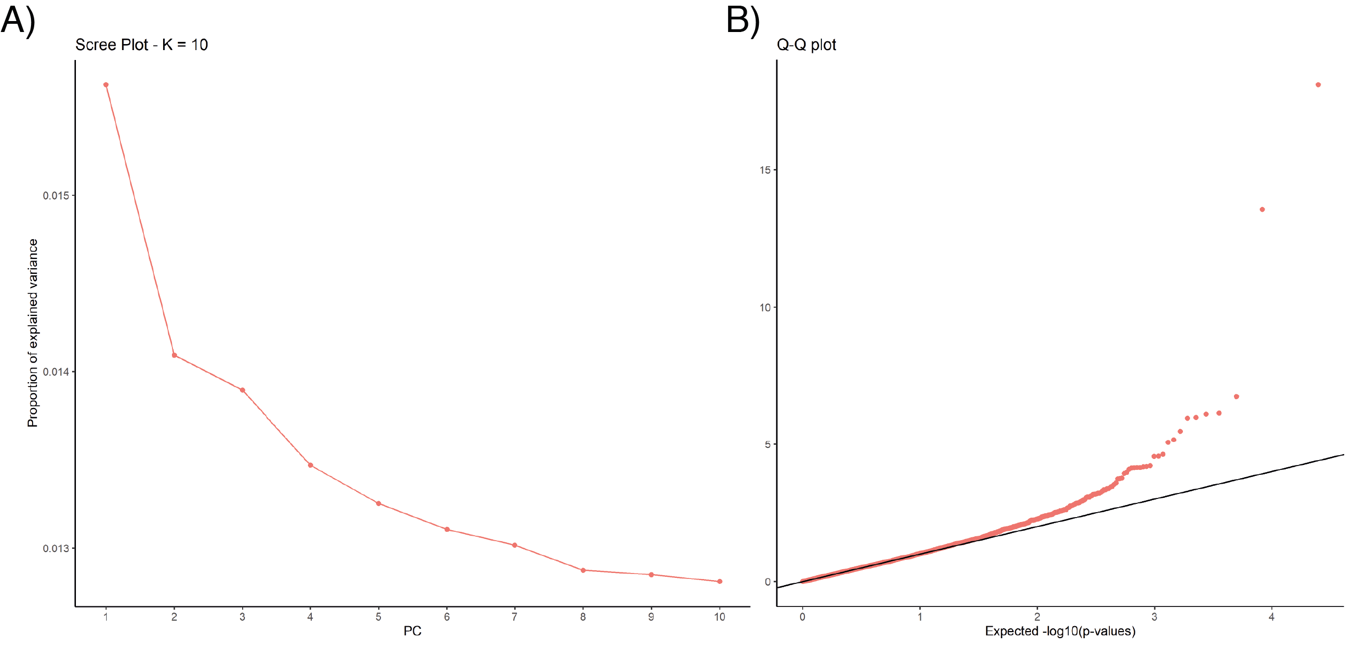


**Figure S1**. Principal Component Analysis of genomic data. A) Scree plot of the proportion of total variability explained by the first 10 PCs of the data. B) Q-Qplot of the p-value distribution for SNPs variants (red dots) used in this study, compared to the expectation under a uniform distribution (black line).

**
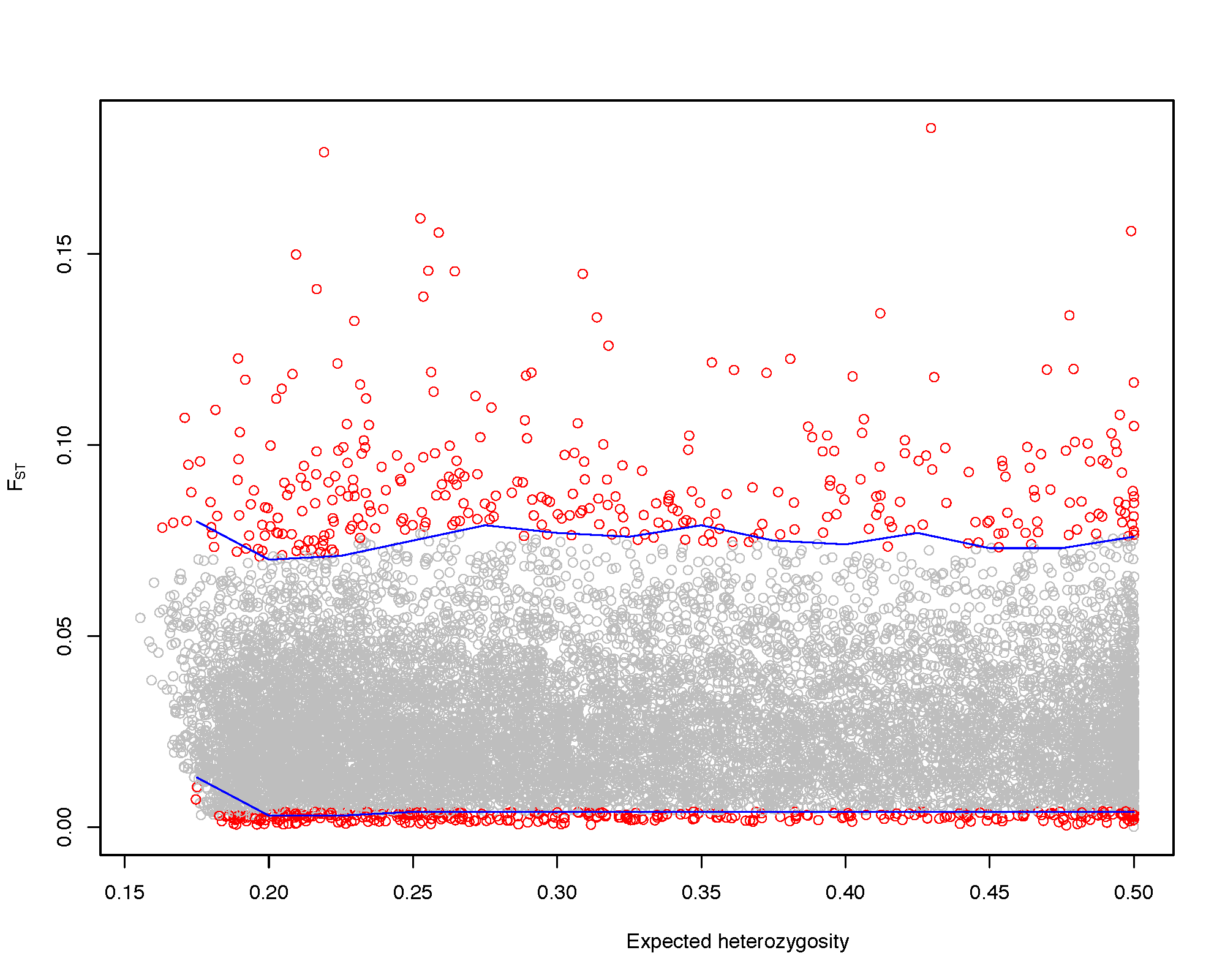
**

**Figure S2.** Plot of *F*_ST_ versus expected heterozygosity to identify potential loci subjected to neutrality and positive selection. Blue lines define the 95% confidence interval (CI). Open gray circles correspond to candidates to neutral markers. Red open circles are candidates for positive selection if they are above the top of 95% CI and for balancing selection if they are below of 95% CI.

**
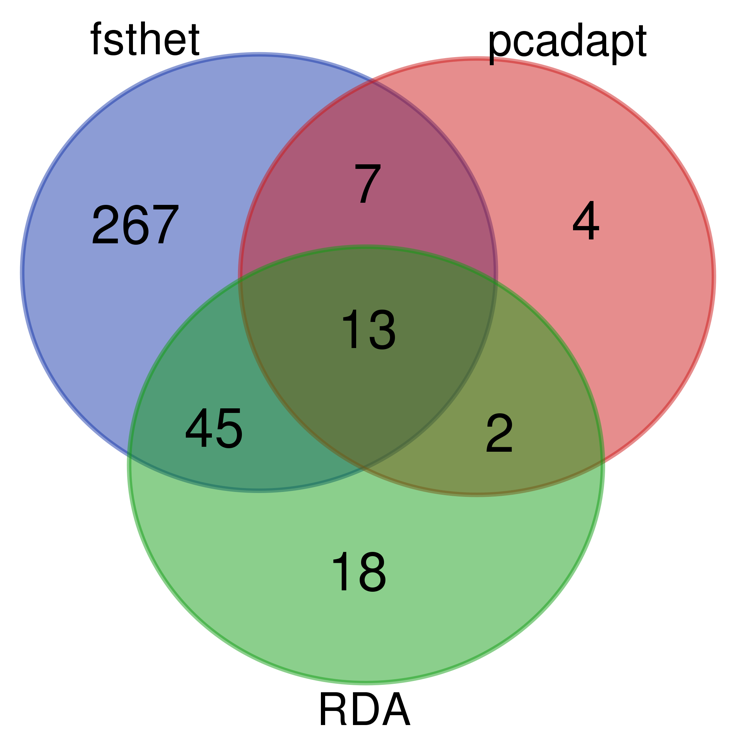
**

**Figure S3**. Venn diagram of genes shared between FSTHET, PCADAPT and RDA analyses for identification of putative adaptive loci.

**
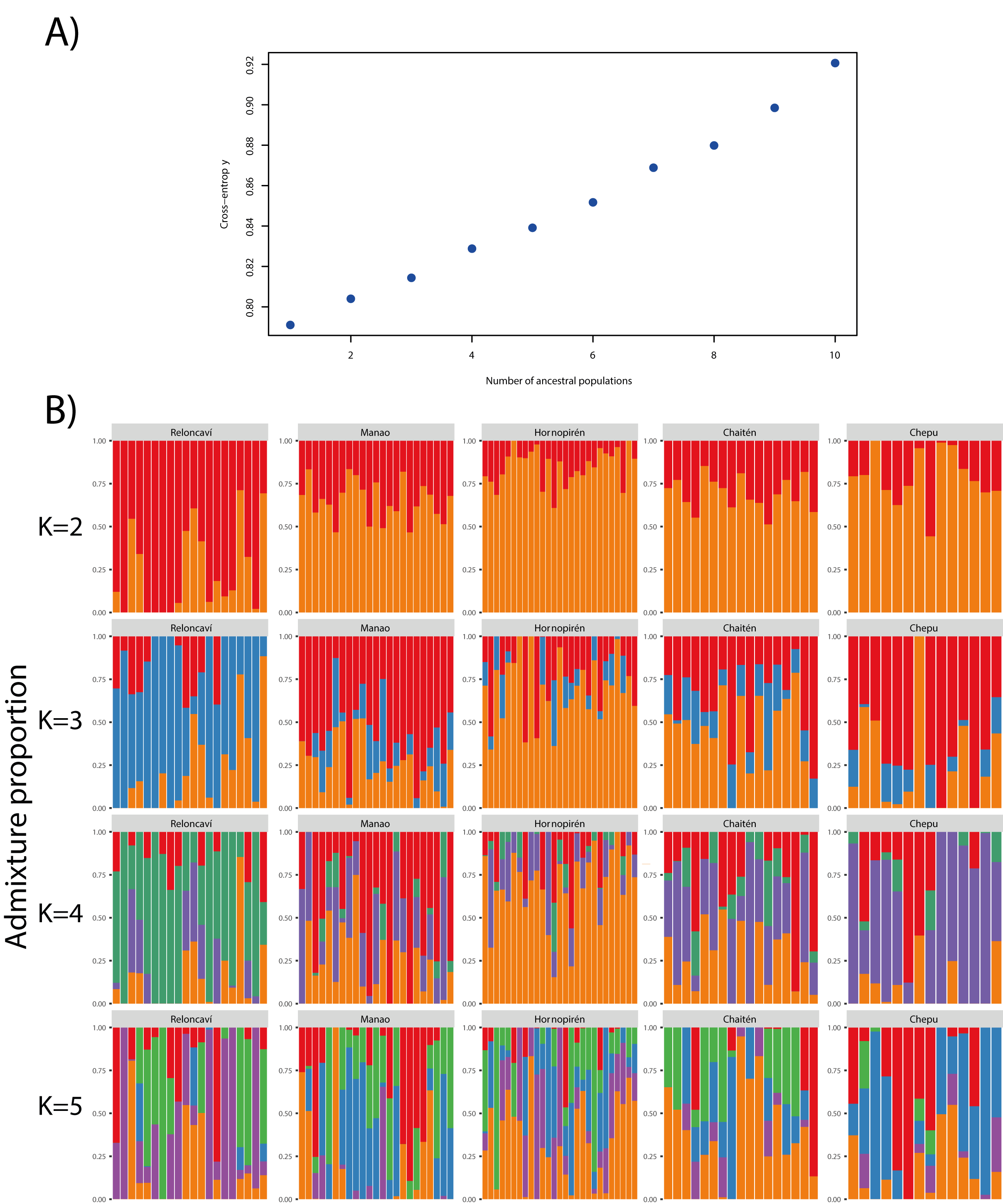
Figure S4.** Values of the cross-entropy criterion and admixture proportions for putative neutral loci (12,026 SNPs). A) The cross-entropy values were obtained for sNMF runs along 10 ancestral populations. B) The admixture proportion results showing the estimated population admixture coefficients (Q) for each individual for K=1 to K=5. Genome is broken into colored segments representing the proportion of that individual's genome derived from each of the K inferred clusters.

**
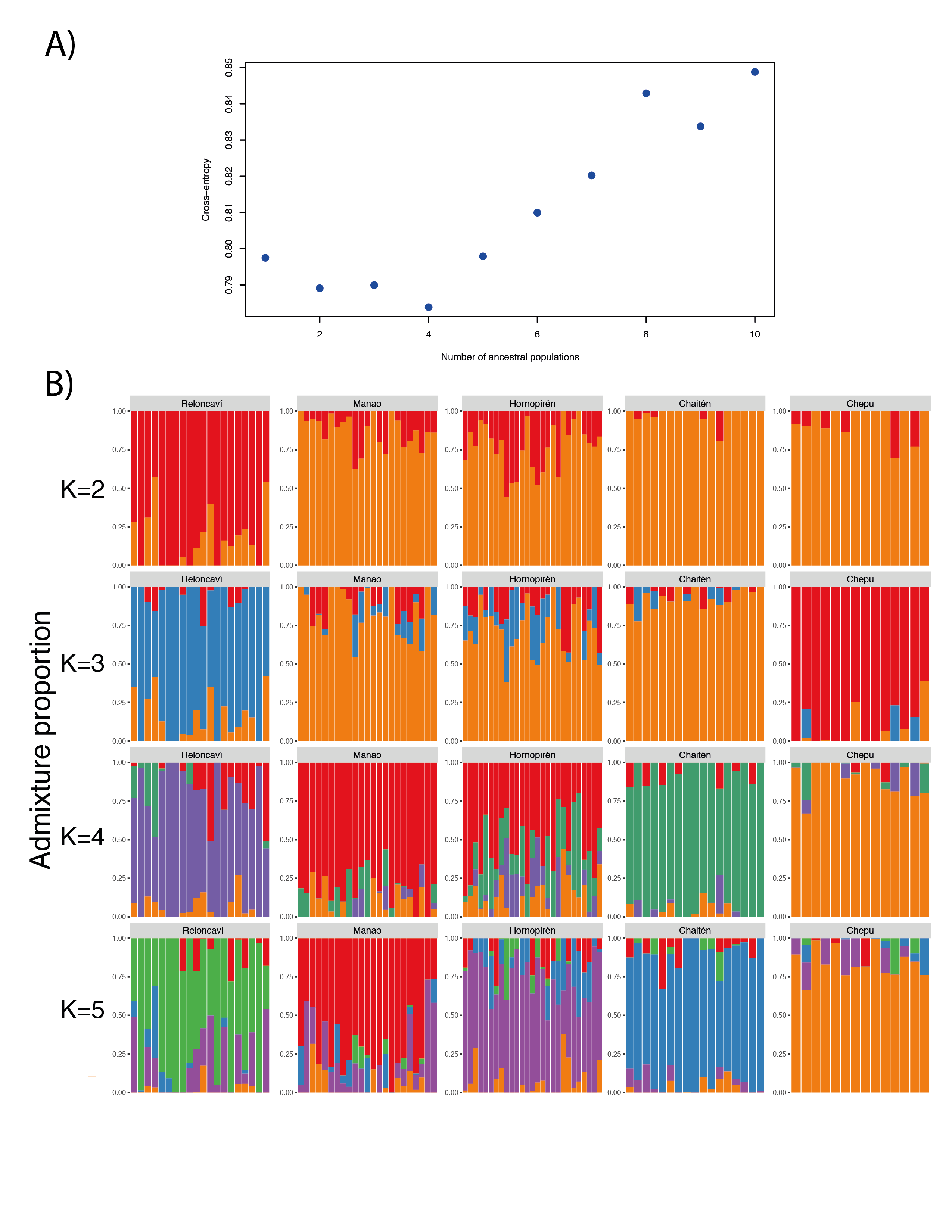
Figure S5.** Values of the cross-entropy criterion and admixture proportions for putative adaptive merged loci for PCADAPT, FSTHET, and RDA analyses (356). A) The cross-entropy values were obtained for sNMF runs along 10 ancestral populations. B) The admixture proportion results showing the estimated population admixture coefficients (Q) for each individual for K=1 to K=5. Genome is broken into colored segments representing the proportion of that individual's genome derived from each of the K inferred clusters.

**
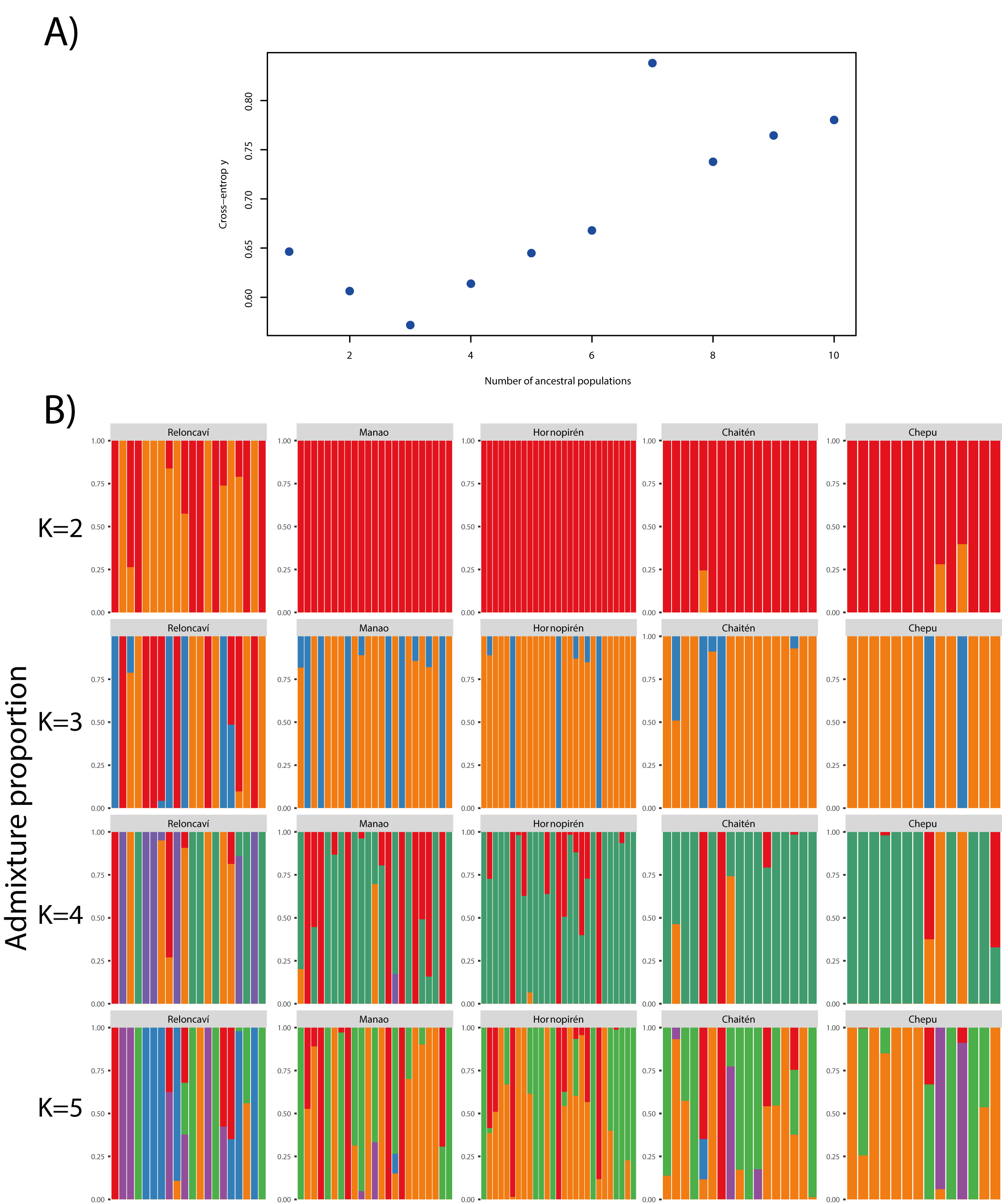
Figure S6.** Values of the cross-entropy criterion and admixture proportions for putative adaptive shared loci among PCADAPT, FSTHET, and RDA analyses. A) The cross-entropy values were obtained for sNMF runs along 10 ancestral populations. B) The admixture proportion results showing the estimated population admixture coefficients (Q) for each individual for K=1 to K=5. Genome is broken into colored segments representing the proportion of that individual's genome derived from each of the K inferred clusters.


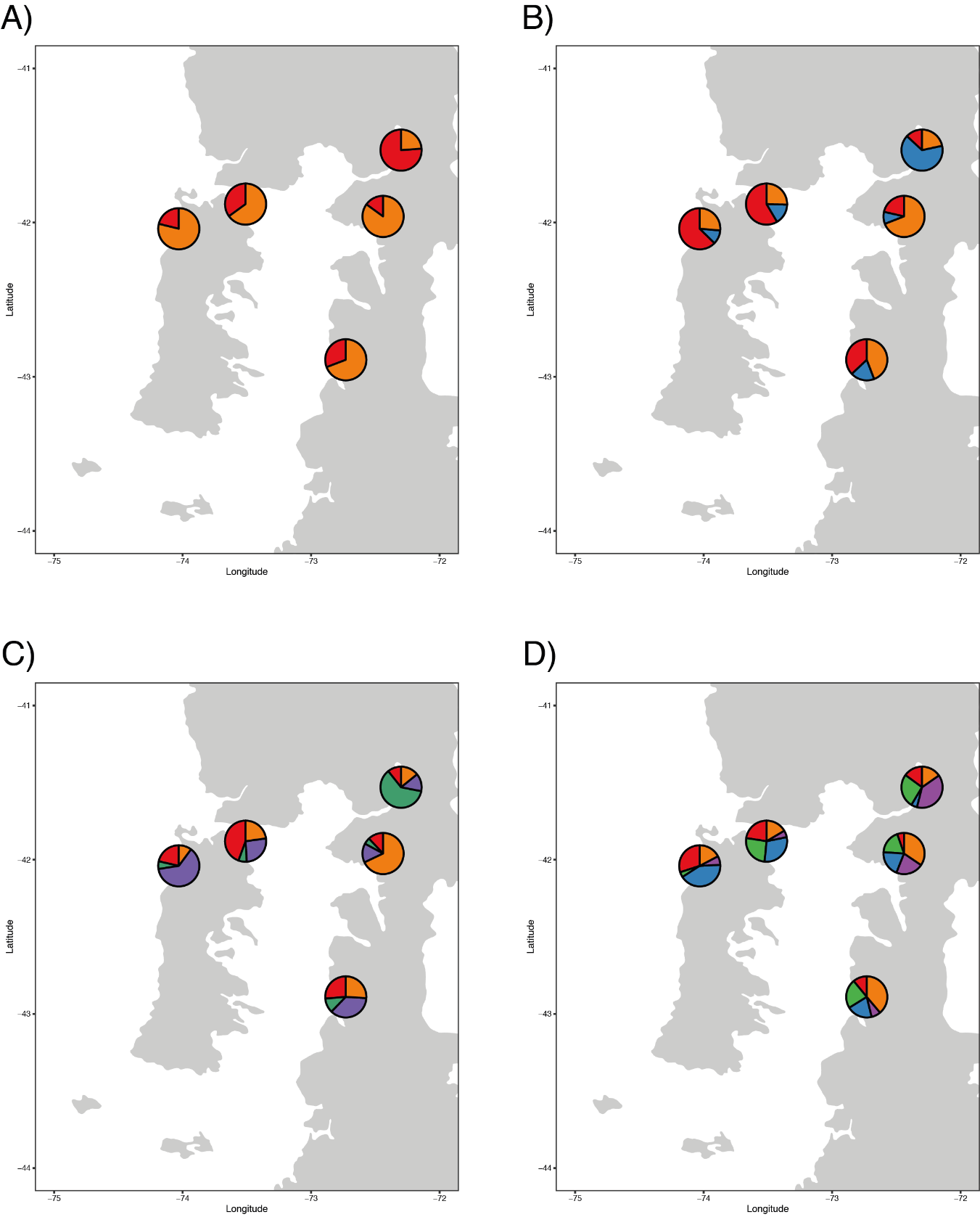


**Figure S7.** Mean admixture proportions by location in putative neutral loci (12,026 SNPs). A) K=1, B) K=2, C) K=3, and D) K=4.


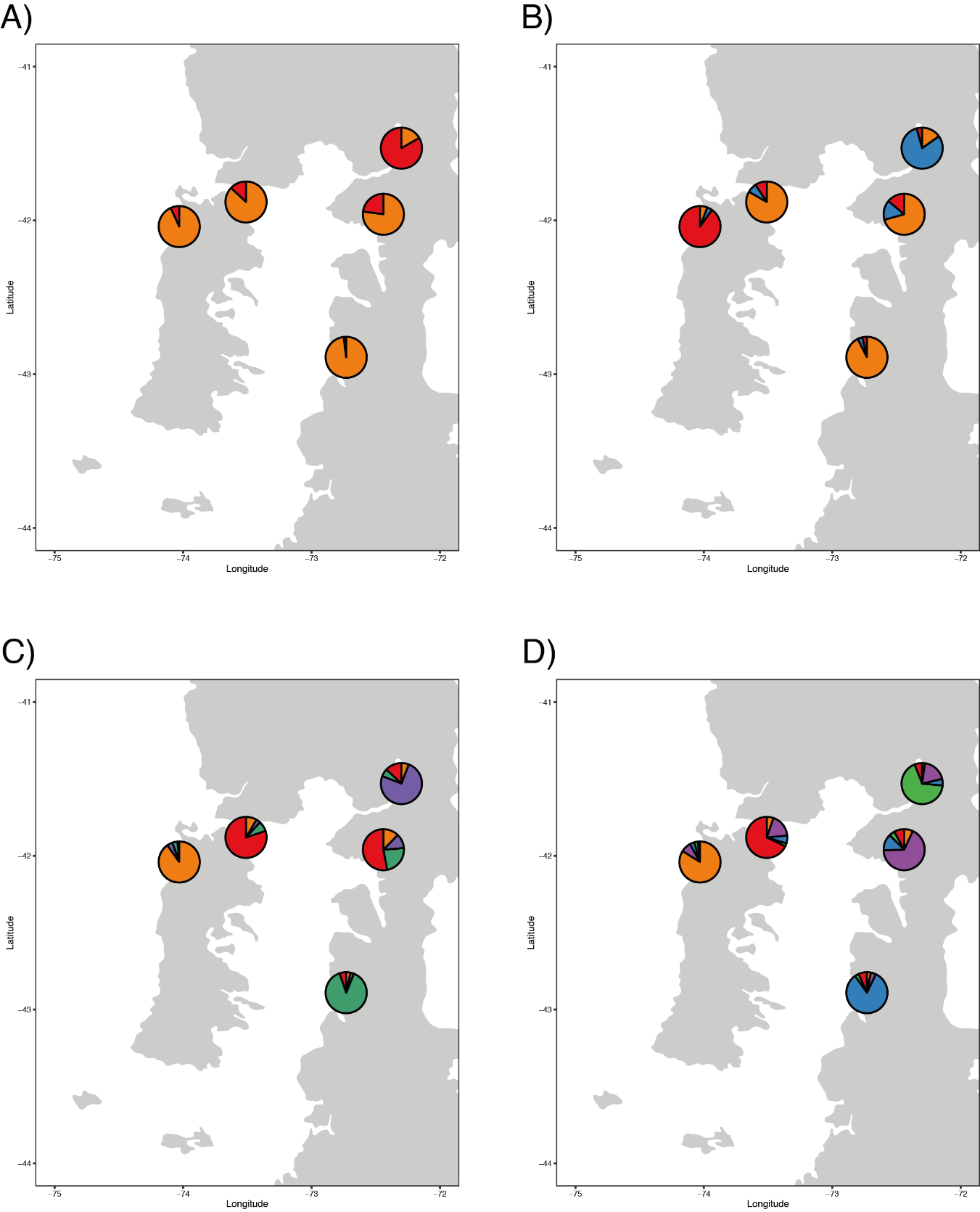


**Figure S8.** Mean admixture proportions by location for putative adaptive loci merged for PCADAPT, FSTHET, and RDA analyses (356 SNPs). A) K=1, B) K=2, C) K=3, and D) K=4.


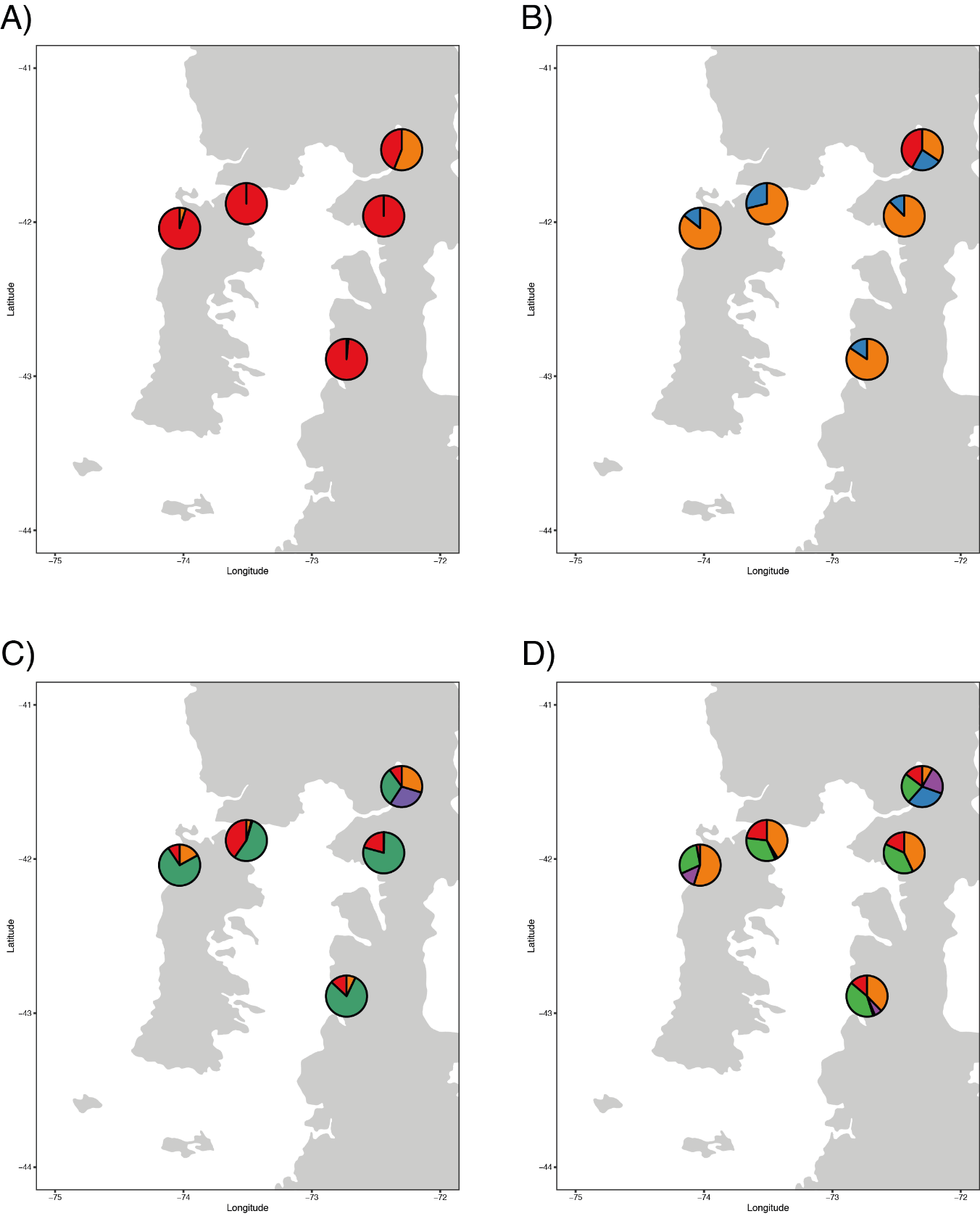


**Figure S9.** Mean admixture proportions by location for putative adaptive loci shared among PCADAPT, FSTHET, and RDA analyses (13 SNPs). A) K=1, B) K=2, C) K=3, and D) K=4.


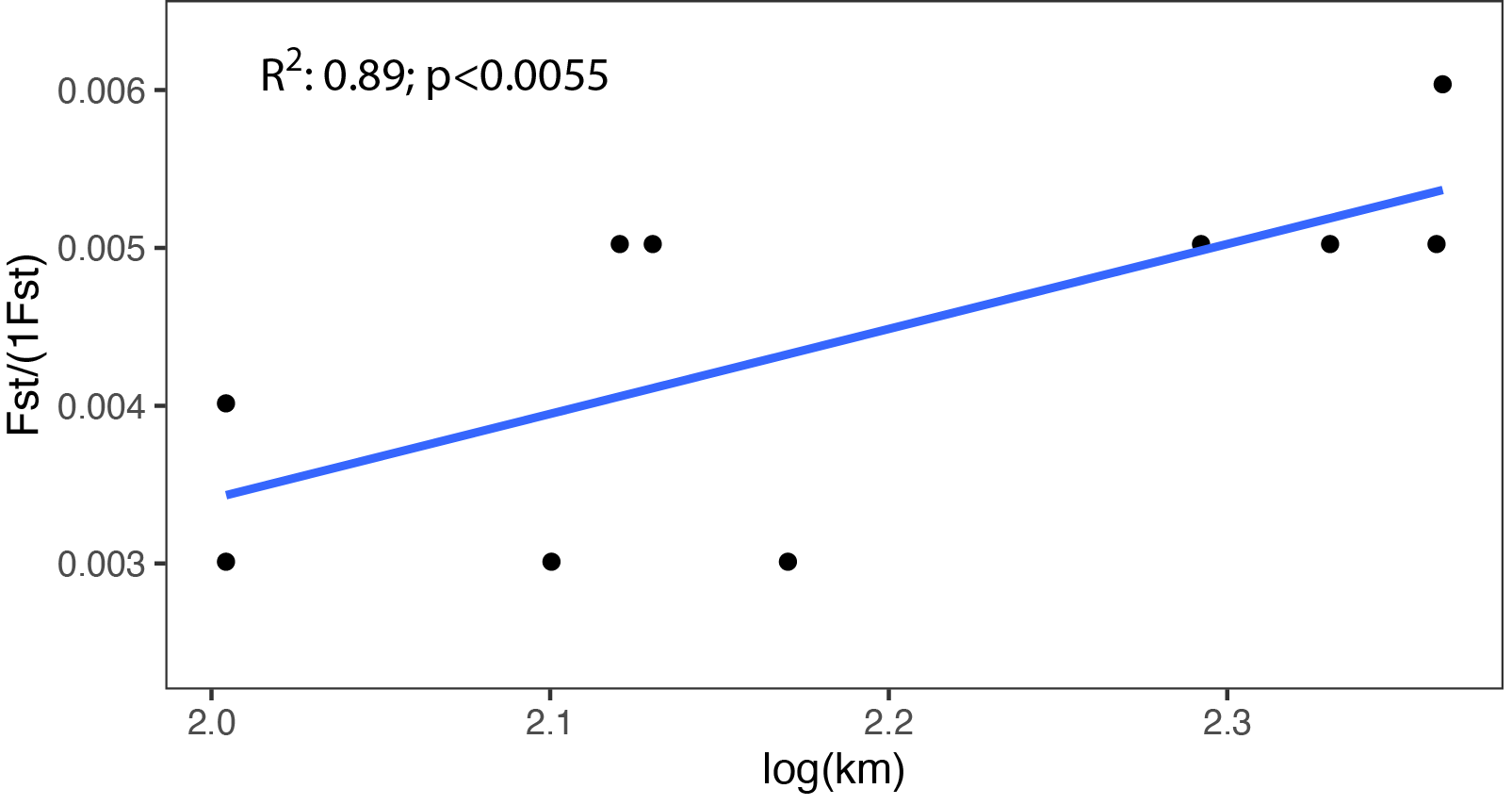


**Figure S10.** Mantel test of isolation by distance on *E*. *maclovinus* populations. Scatter plots of F_ST_ / (1-F_ST_) vs. geographical distances (on a logarithmic scale) for pairwise population comparisons; p-value obtained from 10,000 bootstraps (P<0.0055, R^2^ = 0.89).

**Table S1.** Annotated genes identified by FSTHET, PCADAPT and RDA.

| **tag_snp** | **method** | **variable** | **Gene** | **e-value** | **GO IDs** | **GO Names** |
| --- | --- | --- | --- | --- | --- | --- |
| 625_54 | fsthet | - | Fanconi anemia core complex-associated protein 100 | 1,30E-61 | P:GO:0036297; C:GO:0043240 | P:interstrand cross-link repair; C:Fanconi anaemia nuclear complex |
| 628_87 | fsthet/rda | oxygen | palladin isoform X2 | 4,42E-36 | C:GO:0016021 | C:integral component of membrane |
| 1383_263 | fsthet | - | UDP-glucose:glycoprotein glucosyltransferase 2 isoform X2 | 3,89E-18 | P:GO:0006486; P:GO:0097359; F:GO:0003980; C:GO:0005788 | P:protein glycosylation; P:UDP-glucosylation; F:UDP-glucose:glycoprotein glucosyltransferase activity; C:endoplasmic reticulum lumen |
| 1391_198 | fsthet | - | zinc finger protein Dzip1 isoform X2 | 1,03E-36 | C:GO:0005814 | C:centriole |
| 1586_229 | fsthet | - | DUF1891 domain-containing protein | 1,14E-30 | F:GO:0003677; F:GO:0008168; F:GO:0016706; F:GO:0046872; C:GO:0016021 | F:DNA binding; F:methyltransferase activity; F:2-oxoglutarate-dependent dioxygenase activity; F:metal ion binding; C:integral component of membrane |
| 1701_190 | fsthet/rda | oxygen | protein ELFN1 | 9,91E-56 | C:GO:0016021 | C:integral component of membrane |
| 2067_195 | fsthet | - | sodium channel protein type 2 subunit alpha-like | 2,97E-03 | P:GO:0035725; F:GO:0005248; C:GO:0001518 | P:sodium ion transmembrane transport; F:voltage-gated sodium channel activity; C:voltage-gated sodium channel complex |
| 2306_247 | fsthet | - | sorting nexin-19 isoform X1 | 9,26E-12 | F:GO:0035091; C:GO:0016021 | F:phosphatidylinositol binding; C:integral component of membrane |
| 2393_189 | fsthet | - | delta-1-pyrroline-5-carboxylate synthase-like | 2,85E-02 | P:GO:0006561; P:GO:0008152; P:GO:0008652; P:GO:0016310; P:GO:0055129; F:GO:0000166; F:GO:0003824; F:GO:0004349; F:GO:0004350; F:GO:0005524; F:GO:0016301; F:GO:0016491; F:GO:0016620; F:GO:0016740; C:GO:0005737; C:GO:0005739 | P:proline biosynthetic process; P:metabolic process; P:cellular amino acid biosynthetic process; P:phosphorylation; P:L-proline biosynthetic process; F:nucleotide binding; F:catalytic activity; F:glutamate 5-kinase activity; F:glutamate-5-semialdehyde dehydrogenase activity; F:ATP binding; F:kinase activity; F:oxidoreductase activity; F:oxidoreductase activity, acting on the aldehyde or oxo group of donors, NAD or NADP as acceptor; F:transferase activity; C:cytoplasm; C:mitochondrion |
| 3016_32 | fsthet | - | hypothetical protein EYF80_054166 | 2,84E-02 |  |  |
| 3064_227 | fsthet | - | inositol hexakisphosphate and diphosphoinositol-pentakisphosphate kinase 2 isoform X2 | 1,30E-17 | P:GO:0016310; F:GO:0000827; F:GO:0000829; F:GO:0000832; F:GO:0005524; F:GO:0033857; F:GO:0052723; F:GO:0052724; F:GO:0102092; C:GO:0005829 | P:phosphorylation; F:inositol-1,3,4,5,6-pentakisphosphate kinase activity; F:inositol heptakisphosphate kinase activity; F:inositol hexakisphosphate 5-kinase activity; F:ATP binding; F:diphosphoinositol-pentakisphosphate kinase activity; F:inositol hexakisphosphate 1-kinase activity; F:inositol hexakisphosphate 3-kinase activity; F:5-diphosphoinositol pentakisphosphate 3-kinase activity; C:cytosol |
| 3110_55 | fsthet | - | nesprin-1 isoform X2 | 6,42E-25 | F:GO:0051015; C:GO:0005640; C:GO:0016021; C:GO:0034993 | F:actin filament binding; C:nuclear outer membrane; C:integral component of membrane; C:meiotic nuclear membrane microtubule tethering complex |
| 3270_241 | fsthet | - | ryanodine receptor 3 isoform X2 | 1,64E-07 | P:GO:0051209; F:GO:0005219; F:GO:0005516; C:GO:0016021; C:GO:0033017 | P:release of sequestered calcium ion into cytosol; F:ryanodine-sensitive calcium-release channel activity; F:calmodulin binding; C:integral component of membrane; C:sarcoplasmic reticulum membrane |
| 3713_292 | fsthet | - | protein dopey-1 isoform X1 | 2,85E-44 | P:GO:0006895; P:GO:0015031; C:GO:0005829 | P:Golgi to endosome transport; P:protein transport; C:cytosol |
| 3958_115 | fsthet | - | E3 ubiquitin-protein ligase RING2-A-like isoform X1 | 1,10E-29 | P:GO:0035518; F:GO:0016740; F:GO:0046872; C:GO:0000151; C:GO:0016607 | P:histone H2A monoubiquitination; F:transferase activity; F:metal ion binding; C:ubiquitin ligase complex; C:nuclear speck |
| 4541_19 | fsthet/rda | salinity | A disintegrin and metalloproteinase with thrombospondin motifs 1 | 2,61E-96 | P:GO:0006508; P:GO:0007229; F:GO:0004222; F:GO:0008270; C:GO:0005576; C:GO:0031012 | P:proteolysis; P:integrin-mediated signaling pathway; F:metalloendopeptidase activity; F:zinc ion binding; C:extracellular region; C:extracellular matrix |
| 5541_300 | fsthet | - | rRNA N6-adenosine-methyltransferase ZCCHC4 | 1,93E-01 | P:GO:0031167; P:GO:0032259; F:GO:0003676; F:GO:0008168; F:GO:0008270; F:GO:0008988; F:GO:0016740; F:GO:0046872; C:GO:0005634; C:GO:0005730; C:GO:0005737 | P:rRNA methylation; P:methylation; F:nucleic acid binding; F:methyltransferase activity; F:zinc ion binding; F:rRNA (adenine-N6-)-methyltransferase activity; F:transferase activity; F:metal ion binding; C:nucleus; C:nucleolus; C:cytoplasm |
| 6415_129 | fsthet | - | receptor tyrosine-protein kinase erbB-3a | 8,09E-09 | P:GO:0007169; P:GO:0018108; F:GO:0004714; F:GO:0005524; C:GO:0016021 | P:transmembrane receptor protein tyrosine kinase signaling pathway; P:peptidyl-tyrosine phosphorylation; F:transmembrane receptor protein tyrosine kinase activity; F:ATP binding; C:integral component of membrane |
| 6661_258 | fsthet | - | elongation of very long chain fatty acids protein 5 isoform X1 | 2,04E-04 | C:GO:0016020; C:GO:0016021 | C:membrane; C:integral component of membrane |
| 6997_133 | fsthet | - | GRAM domain-containing protein 1B isoform X4 | 9,25E-13 | C:GO:0005789; C:GO:0005886; C:GO:0016021 | C:endoplasmic reticulum membrane; C:plasma membrane; C:integral component of membrane |
| 7058_269 | fsthet | - | Transformation/transcription domain-associated protein 350/400 kDa PCAF-associated factor | 1,60E-17 | P:GO:0016310; P:GO:0016573; F:GO:0016301; C:GO:0000123 | P:phosphorylation; P:histone acetylation; F:kinase activity; C:histone acetyltransferase complex |
| 7718_164 | fsthet | - | mixed lineage kinase domain-like protein | 2,05E-24 | P:GO:0006468; P:GO:0007166; F:GO:0004672; F:GO:0005524 | P:protein phosphorylation; P:cell surface receptor signaling pathway; F:protein kinase activity; F:ATP binding |
| 7925_185 | fsthet/pcadapt/rda | salinity | cytoplasmic dynein 2 heavy chain 1 isoform X1 | 3,57E-20 | P:GO:0007018; F:GO:0005524; F:GO:0008569; F:GO:0045505; F:GO:0051959; C:GO:0005737; C:GO:0005874; C:GO:0005886; C:GO:0005929; C:GO:0030286 | P:microtubule-based movement; F:ATP binding; F:minus-end-directed microtubule motor activity; F:dynein intermediate chain binding; F:dynein light intermediate chain binding; C:cytoplasm; C:microtubule; C:plasma membrane; C:cilium; C:dynein complex |
| 8354_160 | rda | oxygen | serine-protein kinase ATM | 4,98E-45 | P:GO:0006807; P:GO:0006974; P:GO:0016310; P:GO:0044238; P:GO:0044260; F:GO:0004672 | P:nitrogen compound metabolic process; P:cellular response to DNA damage stimulus; P:phosphorylation; P:primary metabolic process; P:cellular macromolecule metabolic process; F:protein kinase activity |
| 9039_114 | fsthet | - | protein Daple-like | 2,20E-25 | P:GO:0016055; P:GO:0030705; C:GO:0005737 | P:Wnt signaling pathway; P:cytoskeleton-dependent intracellular transport; C:cytoplasm |
| 9460_134 | fsthet/rda | salinity | arylsulfatase I-like | 1,57E-40 | F:GO:0008484 | F:sulfuric ester hydrolase activity |
| 10779_27 | fsthet | - | interleukin-1 receptor accessory protein-like 1 isoform X1 | 2,83E-19 | P:GO:0007165; F:GO:0003953; C:GO:0016021; C:GO:0030424; C:GO:0030425 | P:signal transduction; F:NAD+ nucleosidase activity; C:integral component of membrane; C:axon; C:dendrite |
| 11511_57 | fsthet | - | nuclear factor of activated T-cells 5 isoform X2 | 2,90E-18 | P:GO:0006355; P:GO:0006366; F:GO:0000978; F:GO:0003700; C:GO:0005634 | P:regulation of transcription, DNA-templated; P:transcription by RNA polymerase II; F:RNA polymerase II cis-regulatory region sequence-specific DNA binding; F:DNA-binding transcription factor activity; C:nucleus |
| 11680_284 | fsthet | - | collagen alpha-1(IV) chain | 6,59E-16 | F:GO:0005201; C:GO:0005581; C:GO:0005604; C:GO:0016020 | F:extracellular matrix structural constituent; C:collagen trimer; C:basement membrane; C:membrane |
| 11921_132 | fsthet | - | dynein heavy chain 17, axonemal-like | 1,72E+01 |  |  |
| 12326_20 | fsthet | - | diacylglycerol kinase beta | 1,23E-14 | P:GO:0016310; P:GO:0035556; F:GO:0003951; F:GO:0004143; F:GO:0005509; F:GO:0005524 | P:phosphorylation; P:intracellular signal transduction; F:NAD+ kinase activity; F:diacylglycerol kinase activity; F:calcium ion binding; F:ATP binding |
| 12551_82 | fsthet | - | kinesin-like protein KIFC1 | 3,61E-05 | P:GO:0007018; F:GO:0003777; F:GO:0005524; F:GO:0008017; C:GO:0005874 | P:microtubule-based movement; F:microtubule motor activity; F:ATP binding; F:microtubule binding; C:microtubule |
| 12703_275 | fsthet | - | oocyte zinc finger protein XlCOF7.1-like isoform X3 | 6,64E-14 | F:GO:0003677 | F:DNA binding |
| 12849_63 | fsthet | - | V-set and immunoglobulin domain-containing protein 10-like 2 | 1,21E-50 | C:GO:0016021 | C:integral component of membrane |
| 13538_15 | fsthet/rda | salinity | F-box/LRR-repeat protein 14-like | 6,28E-30 |  |  |
| 13932_275 | fsthet | - | solute carrier family 12 member 5b isoform X2 | 7,92E-17 | P:GO:0007268; P:GO:0071805; P:GO:1902476; F:GO:0015379; C:GO:0005887; C:GO:0045202 | P:chemical synaptic transmission; P:potassium ion transmembrane transport; P:chloride transmembrane transport; F:potassium:chloride symporter activity; C:integral component of plasma membrane; C:synapse |
| 13934_228 | fsthet | - | heterogeneous nuclear ribonucleoprotein Q isoform X3 | 2,61E-01 | P:GO:0000398; P:GO:0001649; P:GO:0006396; P:GO:0006397; P:GO:0006417; P:GO:0008380; P:GO:0017148; P:GO:0070934; P:GO:0071346; F:GO:0003676; F:GO:0003723; F:GO:0003729; F:GO:0005515; F:GO:0048027; C:GO:0005634; C:GO:0005654; C:GO:0005681; C:GO:0005737; C:GO:0005783; C:GO:0016020; C:GO:0043231; C:GO:0070937; C:GO:0071013; C:GO:0071204; C:GO:0097452; C:GO:1990904 | P:mRNA splicing, via spliceosome; P:osteoblast differentiation; P:RNA processing; P:mRNA processing; P:regulation of translation; P:RNA splicing; P:negative regulation of translation; P:CRD-mediated mRNA stabilization; P:cellular response to interferon-gamma; F:nucleic acid binding; F:RNA binding; F:mRNA binding; F:protein binding; F:mRNA 5'-UTR binding; C:nucleus; C:nucleoplasm; C:spliceosomal complex; C:cytoplasm; C:endoplasmic reticulum; C:membrane; C:intracellular membrane-bounded organelle; C:CRD-mediated mRNA stability complex; C:catalytic step 2 spliceosome; C:histone pre-mRNA 3'end processing complex; C:GAIT complex; C:ribonucleoprotein complex |
| 14623_212 | fsthet | - | hypothetical protein F7725_021648, partial | 3,34E-04 | C:GO:0016021 | C:integral component of membrane |
| 14654_131 | fsthet/rda | primary_productivity | transmembrane protease serine 7 | 7,64E-21 | P:GO:0006508; F:GO:0004252; C:GO:0005615; C:GO:0016021 | P:proteolysis; F:serine-type endopeptidase activity; C:extracellular space; C:integral component of membrane |
| 15401_29 | fsthet | - | ROCK1 kinase | 9,54E-19 | P:GO:0006468; F:GO:0004674; F:GO:0005524 | P:protein phosphorylation; F:protein serine/threonine kinase activity; F:ATP binding |
| 16486_78 | fsthet/rda | oxygen | WD repeat domain-containing protein 83 | 3,95E-09 | P:GO:0016310; F:GO:0016301 | P:phosphorylation; F:kinase activity |
| 16588_169 | fsthet | - | calmodulin-regulated spectrin-associated protein 3-like isoform X1 | 1,58E-65 | P:GO:0031175; F:GO:0005516; F:GO:0008017; F:GO:0030507; C:GO:0005737; C:GO:0005874 | P:neuron projection development; F:calmodulin binding; F:microtubule binding; F:spectrin binding; C:cytoplasm; C:microtubule |
| 16840_246 | fsthet/pcadapt/rda | salinity | voltage-dependent L-type calcium channel subunit beta-1 isoform X4 | 9,82E-18 | P:GO:0034765; P:GO:0070588; F:GO:0005245; C:GO:0005891; C:GO:0042383 | P:regulation of ion transmembrane transport; P:calcium ion transmembrane transport; F:voltage-gated calcium channel activity; C:voltage-gated calcium channel complex; C:sarcolemma |
| 17025_67 | fsthet | - | Bardet-Biedl syndrome 4 protein | 3,46E-29 | P:GO:0002009; P:GO:0007368; P:GO:0043473; P:GO:0048598; P:GO:0048731; P:GO:0051649; C:GO:0016020 | P:morphogenesis of an epithelium; P:determination of left/right symmetry; P:pigmentation; P:embryonic morphogenesis; P:system development; P:establishment of localization in cell; C:membrane |
| 17404_128 | fsthet | - | transmembrane protein 94 isoform X1 | 1,36E-14 | C:GO:0016020; C:GO:0016021 | C:membrane; C:integral component of membrane |
| 18696_111 | fsthet/rda | salinity | Glutaminase kidney isoform, mitochondrial | 1,03E-20 | P:GO:0006541; F:GO:0004359 | P:glutamine metabolic process; F:glutaminase activity |
| 19173_107 | fsthet | - | vigilin | 1,32E-57 | F:GO:0003723; C:GO:0005634 | F:RNA binding; C:nucleus |
| 19444_63 | fsthet | - | slit homolog 1 protein-like | 2,39E-17 | P:GO:0021602; P:GO:0030154; F:GO:0005509; C:GO:0005576 | P:cranial nerve morphogenesis; P:cell differentiation; F:calcium ion binding; C:extracellular region |
| 19991_37 | fsthet | - | serine/threonine-protein phosphatase 4 regulatory subunit 2-A-like isoform X2 | 6,69E-15 | P:GO:0050790; F:GO:0019888; C:GO:0030289 | P:regulation of catalytic activity; F:protein phosphatase regulator activity; C:protein phosphatase 4 complex |
| 20530_268 | fsthet/rda | primary_productivity | sorting nexin-33 | 6,23E-16 | P:GO:0000281; P:GO:0006897; P:GO:0015031; F:GO:0035091; C:GO:0019898; C:GO:0030659 | P:mitotic cytokinesis; P:endocytosis; P:protein transport; F:phosphatidylinositol binding; C:extrinsic component of membrane; C:cytoplasmic vesicle membrane |
| 22359_59 | fsthet | - | protein FAM181A | 8,79E-73 |  |  |
| 22585_192 | fsthet | - | serine/threonine-protein kinase N2-like | 4,06E-21 | P:GO:0006468; P:GO:0007165; F:GO:0004698; F:GO:0005524 | P:protein phosphorylation; P:signal transduction; F:calcium-dependent protein kinase C activity; F:ATP binding |
| 24305_282 | fsthet/rda | oxygen | hypothetical protein EXN66_Car001731 | 9,30E-01 |  |  |
| 24346_213 | fsthet | - | sodium- and chloride-dependent GABA transporter ine | 1,24E-24 | P:GO:0055085; F:GO:0015293; C:GO:0016021 | P:transmembrane transport; F:symporter activity; C:integral component of membrane |
| 24498_54 | rda | primary_productivity | unconventional myosin-XV | 2,95E-36 | P:GO:0007605; F:GO:0003774; F:GO:0003779; F:GO:0005524; C:GO:0005737; C:GO:0016459 | P:sensory perception of sound; F:cytoskeletal motor activity; F:actin binding; F:ATP binding; C:cytoplasm; C:myosin complex |
| 24740_167 | fsthet | - | kinesin-like protein KIF26A isoform X2 | 2,72E-03 | P:GO:0007018; F:GO:0000166; F:GO:0003774; F:GO:0003777; F:GO:0005524; F:GO:0008017 | P:microtubule-based movement; F:nucleotide binding; F:cytoskeletal motor activity; F:microtubule motor activity; F:ATP binding; F:microtubule binding |
| 25086_201 | fsthet | - | G-protein coupled receptor 143 | 2,95E-22 | P:GO:0007166; P:GO:0007186; F:GO:0004930; F:GO:0035240; F:GO:0072544; F:GO:0072545; C:GO:0016021 | P:cell surface receptor signaling pathway; P:G protein-coupled receptor signaling pathway; F:G protein-coupled receptor activity; F:dopamine binding; F:L-DOPA binding; F:tyrosine binding; C:integral component of membrane |
| 28663_209 | fsthet | - | probable tRNA (uracil-O(2)-)-methyltransferase | 3,76E-03 | P:GO:0030488; F:GO:0046872; F:GO:0052665; C:GO:0005737 | P:tRNA methylation; F:metal ion binding; F:tRNA (uracil-2'-O-)-methyltransferase activity; C:cytoplasm |
| 29909_69 | fsthet/rda | salinity | cell division cycle protein 20 homolog B-like | 4,42E-15 | P:GO:0051301; P:GO:1904668; F:GO:0010997; F:GO:0097027 | P:cell division; P:positive regulation of ubiquitin protein ligase activity; F:anaphase-promoting complex binding; F:ubiquitin-protein transferase activator activity |
| 30060_207 | fsthet | - | long-chain fatty acid transport protein 4 | 7,86E-02 | P:GO:0001676; P:GO:0015908; F:GO:0004467; F:GO:0031957; C:GO:0016021 | P:long-chain fatty acid metabolic process; P:fatty acid transport; F:long-chain fatty acid-CoA ligase activity; F:very long-chain fatty acid-CoA ligase activity; C:integral component of membrane |
| 32607_129 | fsthet | - | sorting nexin-33 | 1,34E-10 | P:GO:0000281; P:GO:0006897; P:GO:0015031; F:GO:0035091; C:GO:0019898; C:GO:0030659 | P:mitotic cytokinesis; P:endocytosis; P:protein transport; F:phosphatidylinositol binding; C:extrinsic component of membrane; C:cytoplasmic vesicle membrane |
| 33000_96 | fsthet | - | numb-like protein | 3,56E-02 | P:GO:0050769 | P:positive regulation of neurogenesis |
| 34162_267 | fsthet | - | glutamyl aminopeptidase | 3,19E-14 | P:GO:0006508; F:GO:0008270; F:GO:0070006; C:GO:0005886; C:GO:0016021 | P:proteolysis; F:zinc ion binding; F:metalloaminopeptidase activity; C:plasma membrane; C:integral component of membrane |
| 35543_114 | fsthet | - | engulfment and cell motility protein 1-like | 8,14E+01 | P:GO:0006909; P:GO:0016477; P:GO:0030036 | P:phagocytosis; P:cell migration; P:actin cytoskeleton organization |
| 35677_176 | fsthet | - | alsin-like isoform X4 | 1,70E-31 | P:GO:0050790; F:GO:0005085 | P:regulation of catalytic activity; F:guanyl-nucleotide exchange factor activity |
| 35721_267 | fsthet | - | serine/threonine-protein kinase SBK1 | 3,52E-74 | P:GO:0006468; F:GO:0004674; F:GO:0005524; F:GO:0106310; C:GO:0005737 | P:protein phosphorylation; F:protein serine/threonine kinase activity; F:ATP binding; F:protein serine kinase activity; C:cytoplasm |
| 35753_46 | fsthet | - | C-Jun-amino-terminal kinase-interacting protein 4 isoform X1 | 2,51E-29 | P:GO:0016310; P:GO:0043410; F:GO:0005078; F:GO:0016301; C:GO:0005737 | P:phosphorylation; P:positive regulation of MAPK cascade; F:MAP-kinase scaffold activity; F:kinase activity; C:cytoplasm |
| 35787_48 | fsthet/pcadapt/rda | salinity | inosine-uridine preferring nucleoside hydrolase-like | 2,44E-26 | F:GO:0016787; C:GO:0016021 | F:hydrolase activity; C:integral component of membrane |
| 36013_49 | fsthet | - | SUN domain-containing ossification factor-like isoform X3 | 1,91E-59 | C:GO:0016021 | C:integral component of membrane |
| 36392_216 | fsthet | - | ephrin type-B receptor 4a | 9,58E-73 | P:GO:0018108; P:GO:0048013; F:GO:0005003; F:GO:0005524; C:GO:0005887 | P:peptidyl-tyrosine phosphorylation; P:ephrin receptor signaling pathway; F:ephrin receptor activity; F:ATP binding; C:integral component of plasma membrane |
| 36521_151 | fsthet | - | laminin subunit beta-2 | 5,97E-22 | C:GO:0005604; C:GO:0016020 | C:basement membrane; C:membrane |
| 36750_142 | fsthet | - | regulator of nonsense transcripts 1 homolog | 1,51E-36 | P:GO:0000184; F:GO:0003677; F:GO:0003723; F:GO:0003724; F:GO:0005524; F:GO:0008270; F:GO:0016787; C:GO:0005737 | P:nuclear-transcribed mRNA catabolic process, nonsense-mediated decay; F:DNA binding; F:RNA binding; F:RNA helicase activity; F:ATP binding; F:zinc ion binding; F:hydrolase activity; C:cytoplasm |
| 37230_128 | fsthet/rda | velocity | Elongation factor-like GTPase 1 | 1,52E-21 | P:GO:0006414; F:GO:0003746; F:GO:0003924; F:GO:0005525 | P:translational elongation; F:translation elongation factor activity; F:GTPase activity; F:GTP binding |
| 37292_246 | fsthet | - | inositol-tetrakisphosphate 1-kinase | 1,33E-23 | P:GO:0016310; P:GO:0032957; F:GO:0000287; F:GO:0005524; F:GO:0047325; F:GO:0052725; F:GO:0052726 | P:phosphorylation; P:inositol trisphosphate metabolic process; F:magnesium ion binding; F:ATP binding; F:inositol tetrakisphosphate 1-kinase activity; F:inositol-1,3,4-trisphosphate 6-kinase activity; F:inositol-1,3,4-trisphosphate 5-kinase activity |
| 37301_273 | fsthet | - | membralin isoform X1 | 1,10E-08 | C:GO:0016021 | C:integral component of membrane |
| 37471_37 | fsthet | - | probable E3 ubiquitin-protein ligase MID2 | 9,05E-32 | F:GO:0046872 | F:metal ion binding |
| 37784_53 | fsthet | - | G2/M phase-specific E3 ubiquitin-protein ligase-like isoform X2 | 1,16E-11 | P:GO:0016567; F:GO:0004842 | P:protein ubiquitination; F:ubiquitin-protein transferase activity |
| 38141_72 | fsthet | - | general transcription factor 3C polypeptide 3 | 5,16E-45 | P:GO:0006383 | P:transcription by RNA polymerase III |
| 38282_134 | fsthet | - | cadherin-13 | 7,45E-15 | P:GO:0007156; F:GO:0005509; F:GO:0042803; C:GO:0005886; C:GO:0031225 | P:homophilic cell adhesion via plasma membrane adhesion molecules; F:calcium ion binding; F:protein homodimerization activity; C:plasma membrane; C:anchored component of membrane |
| 38436_32 | fsthet | - | niban-like protein 1 | 4,58E-12 |  |  |
| 38801_269 | fsthet | - | BRISC complex subunit Abraxas 2 isoform X2 | 5,54E-04 | C:GO:0005634 | C:nucleus |
| 38811_127 | fsthet | - | receptor-type tyrosine-protein phosphatase-like N | 4,34E-29 | P:GO:0035335; F:GO:0004725; C:GO:0016021; C:GO:0030141; C:GO:0030658; C:GO:0045202 | P:peptidyl-tyrosine dephosphorylation; F:protein tyrosine phosphatase activity; C:integral component of membrane; C:secretory granule; C:transport vesicle membrane; C:synapse |
| 39028_141 | fsthet | - | PREDICTED: palmdelphin-like | 3,21E+00 |  |  |
| 39328_98 | fsthet | - | tyrosine-protein kinase Mer | 1,21E-21 | F:GO:0016301 | F:kinase activity |
| 39369_278 | fsthet | - | probable D-lactate dehydrogenase, mitochondrial | 6,13E-06 | F:GO:0016491; F:GO:0071949 | F:oxidoreductase activity; F:FAD binding |
| 39416_266 | fsthet | - | WT1 transcription factor b isoform X1 | 2,65E-07 | P:GO:0006355; F:GO:0003723; F:GO:0046872; C:GO:0005737; C:GO:0016607 | P:regulation of transcription, DNA-templated; F:RNA binding; F:metal ion binding; C:cytoplasm; C:nuclear speck |
| 39451_177 | fsthet | - | apolipoprotein L3-like | 8,08E-25 | C:GO:0110165 | C:cellular anatomical entity |
| 40652_244 | fsthet/pcadapt/rda | oxygen | moesin a isoform X1 | 7,47E-21 | F:GO:0003779; C:GO:0005737; C:GO:0005856; C:GO:0005886 | F:actin binding; C:cytoplasm; C:cytoskeleton; C:plasma membrane |
| 40664_219 | fsthet | - | probable tRNA (uracil-O(2)-)-methyltransferase | 2,52E-18 | P:GO:0030488; F:GO:0046872; F:GO:0052665; C:GO:0005737 | P:tRNA methylation; F:metal ion binding; F:tRNA (uracil-2'-O-)-methyltransferase activity; C:cytoplasm |
| 43665_263 | pcadapt | - | tonsoku-like protein | 3,33E-16 |  |  |
| 46061_108 | fsthet | - | transient receptor potential cation channel subfamily M member 6 isoform X1 | 1,91E+00 | P:GO:0006468; P:GO:0006811; P:GO:0006812; P:GO:0006816; P:GO:0016310; P:GO:0051262; P:GO:0055085; P:GO:0070588; F:GO:0004674; F:GO:0004674; F:GO:0005216; F:GO:0005261; F:GO:0005262; F:GO:0005524; F:GO:0016301; F:GO:0016740; F:GO:0106310; C:GO:0016020; C:GO:0016021 | P:protein phosphorylation; P:ion transport; P:cation transport; P:calcium ion transport; P:phosphorylation; P:protein tetramerization; P:transmembrane transport; P:calcium ion transmembrane transport; F:protein serine/threonine kinase activity; F:protein serine/threonine kinase activity; F:ion channel activity; F:cation channel activity; F:calcium channel activity; F:ATP binding; F:kinase activity; F:transferase activity; F:protein serine kinase activity; C:membrane; C:integral component of membrane |
| 46290_228 | rda | primary_productivity | protein Daple isoform X1 | 3,13E-08 | P:GO:0016055; P:GO:0030705; C:GO:0005737 | P:Wnt signaling pathway; P:cytoskeleton-dependent intracellular transport; C:cytoplasm |
| 48957_108 | fsthet | - | protein tyrosine kinase 2aa isoform X12 | 5,17E-07 | P:GO:0007172; P:GO:0018108; F:GO:0004714; F:GO:0004715; F:GO:0005524; C:GO:0005856; C:GO:0005886; C:GO:0005925; C:GO:0042995 | P:signal complex assembly; P:peptidyl-tyrosine phosphorylation; F:transmembrane receptor protein tyrosine kinase activity; F:non-membrane spanning protein tyrosine kinase activity; F:ATP binding; C:cytoskeleton; C:plasma membrane; C:focal adhesion; C:cell projection |
| 50000_74 | fsthet | - | E3 ubiquitin-protein ligase UBR4 isoform X1 | 1,23E-29 |  |  |
| 51093_144 | fsthet | - | Myosin-M heavy chain | 5,01E-15 | P:GO:0050790; F:GO:0005085 | P:regulation of catalytic activity; F:guanyl-nucleotide exchange factor activity |
| 51147_143 | fsthet | - | atrial natriuretic peptide receptor 1-like isoform X1 | 6,13E-03 | P:GO:0006182; P:GO:0006468; P:GO:0035556; P:GO:0071678; F:GO:0004383; F:GO:0004672; F:GO:0005524; C:GO:0016021 | P:cGMP biosynthetic process; P:protein phosphorylation; P:intracellular signal transduction; P:olfactory bulb axon guidance; F:guanylate cyclase activity; F:protein kinase activity; F:ATP binding; C:integral component of membrane |
| 51481_248 | fsthet/rda | salinity | kinesin-like protein KIF20A | 2,40E-16 | F:GO:0000166; F:GO:0003774 | F:nucleotide binding; F:cytoskeletal motor activity |
| 52015_322 | fsthet | - | protein phosphatase 1 regulatory subunit 21 isoform X2 | 7,14E-35 | C:GO:0005769 | C:early endosome |
| 52259_167 | fsthet | - | transmembrane protease serine 6 | 4,79E-22 | P:GO:0006508; F:GO:0004252; C:GO:0005615; C:GO:0016021 | P:proteolysis; F:serine-type endopeptidase activity; C:extracellular space; C:integral component of membrane |
| 52722_68 | fsthet | - | EH domain-containing protein 4-like | 2,33E-22 | P:GO:0032456; F:GO:0005509; F:GO:0005525; C:GO:0005886 | P:endocytic recycling; F:calcium ion binding; F:GTP binding; C:plasma membrane |
| 54530_229 | fsthet | - | protocadherin Fat 3 | 5,52E-18 | P:GO:0007156; F:GO:0005509; C:GO:0005886; C:GO:0016021 | P:homophilic cell adhesion via plasma membrane adhesion molecules; F:calcium ion binding; C:plasma membrane; C:integral component of membrane |
| 54656_69 | fsthet/pcadapt/rda | oxygen | zinc finger protein 395b isoform X1 | 1,04E-08 | C:GO:0016021 | C:integral component of membrane |
| 54783_34 | fsthet/rda | salinity | unconventional myosin-XV | 1,85E-72 | P:GO:0006836; P:GO:0007605; P:GO:0015812; P:GO:0055085; F:GO:0003774; F:GO:0003779; F:GO:0005332; F:GO:0005524; C:GO:0005887; C:GO:0016459 | P:neurotransmitter transport; P:sensory perception of sound; P:gamma-aminobutyric acid transport; P:transmembrane transport; F:cytoskeletal motor activity; F:actin binding; F:gamma-aminobutyric acid:sodium symporter activity; F:ATP binding; C:integral component of plasma membrane; C:myosin complex |
| 55823_179 | fsthet | - | AP2A factor | 1,74E-03 | P:GO:0006355; F:GO:0003677; F:GO:0003700; C:GO:0005634 | P:regulation of transcription, DNA-templated; F:DNA binding; F:DNA-binding transcription factor activity; C:nucleus |
| 56000_161 | fsthet | - | Hypothetical protein SMAX5B_007563, partial | 4,82E-11 |  |  |
| 56058_128 | fsthet | - | probable 28S rRNA (cytosine(4447)-C(5))-methyltransferase | 4,51E-24 | P:GO:0001510; P:GO:0006396; P:GO:0042254; F:GO:0003723; F:GO:0008757; C:GO:0005730 | P:RNA methylation; P:RNA processing; P:ribosome biogenesis; F:RNA binding; F:S-adenosylmethionine-dependent methyltransferase activity; C:nucleolus |
| 56935_112 | fsthet | - | zinc finger protein 277 | 7,67E-26 |  |  |
| 58007_253 | fsthet | - | transcriptional repressor NF-X1 | 2,21E-28 | F:GO:0005488 | F:binding |
| 58269_51 | pcadapt | - | glycerol-3-phosphate dehydrogenase 1-like protein | 1,91E-07 | P:GO:0005975; P:GO:0046168; F:GO:0004367; F:GO:0051287; C:GO:0009331 | P:carbohydrate metabolic process; P:glycerol-3-phosphate catabolic process; F:glycerol-3-phosphate dehydrogenase [NAD+] activity; F:NAD binding; C:glycerol-3-phosphate dehydrogenase complex |
| 58904_105 | fsthet/rda | salinity | ubiquitin carboxyl-terminal hydrolase 7 | 3,24E-07 | P:GO:0006511; P:GO:0016579; F:GO:0004843 | P:ubiquitin-dependent protein catabolic process; P:protein deubiquitination; F:thiol-dependent deubiquitinase |
| 59066_65 | fsthet | - | dystrophin isoform X3 | 1,06E-23 | C:GO:0110165 | C:cellular anatomical entity |
| 59244_124 | fsthet | - | NLR family CARD domain-containing protein 3-like | 4,83E-07 | P:GO:0000012; P:GO:0006284; P:GO:0006303; F:GO:0003684; C:GO:0005634 | P:single strand break repair; P:base-excision repair; P:double-strand break repair via nonhomologous end joining; F:damaged DNA binding; C:nucleus |
| 60673_125 | fsthet | - | low-density lipoprotein receptor-related protein 2 | 4,74E-15 | P:GO:0006897; F:GO:0005509; C:GO:0016021 | P:endocytosis; F:calcium ion binding; C:integral component of membrane |
| 60804_182 | fsthet | - | erythroid differentiation-related factor 1 isoform X2 | 4,47E-22 |  |  |
| 60991_249 | fsthet | - | nucleoporin NUP188 homolog | 2,43E-49 | P:GO:0006913; P:GO:0015031; F:GO:0017056; C:GO:0005643 | P:nucleocytoplasmic transport; P:protein transport; F:structural constituent of nuclear pore; C:nuclear pore |
| 61195_37 | fsthet | - | eukaryotic translation initiation factor 3 subunit B | 9,44E-17 | P:GO:0001732; F:GO:0003743; F:GO:0031369; C:GO:0005852; C:GO:0016282; C:GO:0033290 | P:formation of cytoplasmic translation initiation complex; F:translation initiation factor activity; F:translation initiation factor binding; C:eukaryotic translation initiation factor 3 complex; C:eukaryotic 43S preinitiation complex; C:eukaryotic 48S preinitiation complex |
| 61610_23 | fsthet | - | Frizzled-4 | 3,36E-17 | P:GO:0016055; F:GO:0042813; C:GO:0016021 | P:Wnt signaling pathway; F:Wnt-activated receptor activity; C:integral component of membrane |
| 61897_186 | fsthet/pcadapt/rda | oxygen | serine/threonine-protein kinase SMG1 | 6,65E-16 | P:GO:0000184; P:GO:0006468; F:GO:0004674; F:GO:0005524; F:GO:0106310 | P:nuclear-transcribed mRNA catabolic process, nonsense-mediated decay; P:protein phosphorylation; F:protein serine/threonine kinase activity; F:ATP binding; F:protein serine kinase activity |
| 62204_172 | fsthet | - | collagen alpha-1(IV) chain | 4,43E-02 | F:GO:0005201; C:GO:0005581; C:GO:0005604; C:GO:0016020 | F:extracellular matrix structural constituent; C:collagen trimer; C:basement membrane; C:membrane |
| 64536_111 | fsthet | - | aftiphilin isoform X2 | 4,46E-66 |  |  |
| 64677_253 | fsthet/pcadapt/rda | oxygen | apoptosis-inducing factor 1, mitochondrial isoform X15 | 4,25E-05 | P:GO:0006915; F:GO:0016491; F:GO:0046983; F:GO:0050660; C:GO:0005739 | P:apoptotic process; F:oxidoreductase activity; F:protein dimerization activity; F:flavin adenine dinucleotide binding; C:mitochondrion |
| 64780_282 | fsthet/pcadapt/rda | oxygen | sestrin-1 isoform X1 | 2,38E-29 | P:GO:0007368; P:GO:1901031; C:GO:0005634; C:GO:0005737 | P:determination of left/right symmetry; P:regulation of response to reactive oxygen species; C:nucleus; C:cytoplasm |
| 64848_72 | fsthet/rda | velocity | R2DM Retrovirus-related Pol polyprotein from type II retrotransposable element | 2,09E-20 | P:GO:0006518; F:GO:0004504; F:GO:0004598; F:GO:0005507; C:GO:0016021; C:GO:0030658 | P:peptide metabolic process; F:peptidylglycine monooxygenase activity; F:peptidylamidoglycolate lyase activity; F:copper ion binding; C:integral component of membrane; C:transport vesicle membrane |
| 75211_204 | fsthet | - | proline-, glutamic acid- and leucine-rich protein 1 | 1,59E-13 | C:GO:0005634; C:GO:0005737 | C:nucleus; C:cytoplasm |
